# STING is required for host defense against neuropathological West Nile virus infection

**DOI:** 10.1371/journal.ppat.1007899

**Published:** 2019-08-15

**Authors:** Kathryn McGuckin Wuertz, Piper M. Treuting, Emily A. Hemann, Katharina Esser-Nobis, Annelise G. Snyder, Jessica B. Graham, Brian P. Daniels, Courtney Wilkins, Jessica M. Snyder, Kathleen M. Voss, Andrew Oberst, Jennifer Lund, Michael Gale

**Affiliations:** 1 Department of Global Health, University of Washington, Seattle, WA, United States of America; 2 Department of Immunology, University of Washington, Seattle, WA, United States of America; 3 Center for Innate Immunity and Immune Disease, University of Washington, Seattle, WA, United States of America; 4 Department of Defense; United States Army Medical Department, San Antonio, TX, United States of America; 5 Department of Comparative Medicine, University of Washington, Seattle, WA, United States of America; 6 Vaccine and Infectious Disease Division, Fred Hutchinson Cancer Research Center, Seattle, WA, United States of America; 7 Department of Cell Biology and Neuroscience, Rutgers University, Piscataway, NJ, United States of America; National Institute of Allergy and Infectious Diseases, UNITED STATES

## Abstract

West Nile Virus (WNV), an emerging and re-emerging RNA virus, is the leading source of arboviral encephalitic morbidity and mortality in the United States. WNV infections are acutely controlled by innate immunity in peripheral tissues outside of the central nervous system (CNS) but WNV can evade the actions of interferon (IFN) to facilitate CNS invasion, causing encephalitis, encephalomyelitis, and death. Recent studies indicate that *ST*imulator of *IN*terferon *G*ene (STING), canonically known for initiating a type I IFN production and innate immune response to cytosolic DNA, is required for host defense against neurotropic RNA viruses. We evaluated the role of STING in host defense to control WNV infection and pathology in a murine model of infection. When challenged with WNV, STING knock out (-/-) mice displayed increased morbidity and mortality compared to wild type (WT) mice. Virologic analysis and assessment of STING activation revealed that STING signaling was not required for control of WNV in the spleen nor was WNV sufficient to mediate canonical STING activation *in vitro*. However, STING-/- mice exhibited a clear trend of increased viral load and virus dissemination in the CNS. We found that STING-/- mice exhibited increased and prolonged neurological signs compared to WT mice. Pathological examination revealed increased lesions, mononuclear cellular infiltration and neuronal death in the CNS of STING-/- mice, with sustained pathology after viral clearance. We found that STING was required in bone marrow derived macrophages for early control of WNV replication and innate immune activation. *In vivo*, STING-/- mice developed an aberrant T cell response in both the spleen and brain during WNV infection that linked with increased and sustained CNS pathology compared to WT mice. Our findings demonstrate that STING plays a critical role in immune programming for the control of neurotropic WNV infection and CNS disease.

## Author summary

In recent years, outbreaks of emerging and re-emerging neuroinvasive West Nile virus (WNV) infection have brought about a critical need to understand host factors that restrict neuropathology and disease. WNV infection in humans typically is either asymptomatic or results in a mild febrile illness, but in some cases virus spreads to the central nervous (CNS) causing a more severe form of neuropathological disease. Previous studies established that both innate and adaptive immune responses are essential for controlling WNV disease and restricting the virus from the CNS. In this study, we examined the role of *St*imulator of *In*terferon *G*enes (STING) in conferring host defense during WNV infection in a murine model. Our studies revealed that STING is essential for restricting pathology in the CNS during WNV infection. Further, STING is required for effective programming of the innate and adaptive immune response to WNV. In the absence of STING, aberrant immune development leads to ineffective viral clearance and immunopathology in the CNS. These studies uncover a critical and previously unidentified role for STING in the restriction of WNV that may have broader implications for a role in conferring host defense against RNA viruses.

## Introduction

Encephalitic *Flavivirus* infections, including West Nile virus (WNV), are ongoing or emerging threats to global health [1–4]. In particular, WNV continues to re-emerge in the Americas, causing neuropathology and death in the most severe cases [3, 5–7]. Since its emergence in the USA in 1999, annual outbreaks of WNV are impacted with fluctuations in neurovirulence attributed to the circulating strain [4–6, 8, 9]. Morbidity and mortality are dramatically increased in years where the circulating strain has enhanced neurovirulence, highlighting the significance of understanding host-pathogen interactions that control neurotropism [5, 10]. An analysis of CDC reports reveals that of all cases reported between 1999–2014, 9% of neurovirulent cases result in death, in contrast to 0.5% of non-neurovirulent WNV cases. Factors that limit WNV neurovirulence are not well understood but are critical to restrict pathology associated with WNV infections [5].

WNV infection in humans most commonly manifests as an asymptomatic or mild febrile illness known as West Nile Fever (WNF) with symptoms that include headache, generalized weakness, rash, fever or myalgia, and in some cases vomiting, diarrhea, joint or eye pain [3, 5–7, 11–13]. While most patients displaying WNF generally display symptoms for days to weeks, in some cases persistent symptoms continue to impact quality of life and cognitive abilities rendering a chronic disease outcome to WNV infection [11]. More serious disease occurs if the virus crosses the blood brain barrier and progress to West Nile Neuroinvasive Disease (WNND) [7]. WNND disease symptoms include meningitis, encephalitis, myelitis marked with acute flaccid paralysis, gastric complications, tremors and Parkinson-like symptoms [7, 11, 14–18]. Patients with WNND can maintain symptoms for weeks to months, with persistent symptoms including chronic fatigue, functional cognitive disorders or neuropsychiatric disabilities and physiological complications, particularly those who exhibited acute flaccid paralysis symptoms during acute infection [7, 11, 18]. Currently no therapeutics or vaccines are available for treatment of WNV infection or neuropathogenesis. Thus, there remains a critical need to understand the virus-host interactions of WNV neurovirulence.

Both the innate and adaptive immune response are required to clear WNV infection and restrict immune mediated pathology [19]. In humans, infection with WNV typically occurs through subcutaneous inoculation from the bite of an infected mosquito. A parallel form of infection using sub-cutaneous challenge of WNV in a mouse model has been shown to replicate the progression, tissue involvement, and pathology of WNV infection that occurs in humans [19–22]. In the mouse model, viral replication occurs at the subcutaneous site of entry followed by infection of the draining lymph node and splenic infection [19]. These processes first trigger innate immune activation in peripheral tissues outside of the central nervous system (CNS) through viral recognition by the RIG-I-like receptors to induce IRF3 activation and the production of types I and III interferon (IFN) [23–26]. Innate (RLR) immune defenses triggered by RLR signaling and IFN actions serve to restrict the tissue tropism of WNV and are essential for protection against neuroinvasion [19, 23, 24, 27–34]. Type I and III IFN are essential to inform the innate and adaptive immune interface to balance development of effective immunity, protect the blood-brain barrier, and limit immune-related pathology in the CNS [19, 23, 24, 35–39]. In particular, type I IFN-dependent cytokine and chemokine signaling cascades are essential for functional development of the cytotoxic CD8+ T cell response, as well as its regulatory T cell (Tregs; FoxP3+ CD4+ T cells) counterpart [24, 36, 37, 39–42]. While CD8+ T cells are required for controlling both peripheral and CNS viral load, CD4+ T cells, specifically Tregs, are essential for preventing symptomatic disease in the CNS [40–43].

The adaptor protein, *St*imulator of *In*terferon *G*enes (STING), has also been implicated in host defense against WNV [44–46]. STING was first described as an essential defense mechanism against both RNA and DNA viruses [47, 48]. Since then, STING has been recognized for its role in responding to cytoplasmic DNA and mediating subsequent innate immune activation and IFN production. However its role in the defense against RNA viruses is poorly understood [47–54]. Intriguingly, multiple RNA viruses, including dengue virus, yellow fever virus, hepatitis C virus and coronaviruses, direct viral evasion strategies to disrupt the STING signaling pathway, reflecting a likely role for STING in host defense against RNA viruses [52]. STING was found to be required for host defense during infection with influenza A virus, as well as dengue virus, a closely related flavivirus to WNV [55–57]. Additionally, during infection with related flavivuses including Japanese encephalitis virus (JEV) and Zika virus, STING deficiency led to increased neuropathology *in vivo* and *in vitro*, suggesting a critical role for STING in CNS defense [58, 59]. The role for STING in the CNS has been implicated in multiple other neurodegenerative diseases including Aicardi-Goutières syndrome, sterile immune mediated CNS pathology and during chronic CNS diseases [14, 16, 60–66].

In this study, we investigated the hypothesis that STING plays a regulatory role in the immune response against WNV, thereby restricting viral neurotropism and neuropathology. We show that STING is essential for host defense against WNV in a mouse *in vivo* model of infection. Clinical and pathological analyses demonstrate a novel role for STING in conferring CNS defense against WNV *in vivo*. We found that tonic levels of type I IFN were decreased in STING-/- bone marrow derived macrophages (BMDM) and linked with increased susceptibility to WNV infection. Following infection, we observed heightened immune responses *in vitro* and *in vivo* concomitant with increased viral load. STING deficiency led to the development of an aberrant adaptive immune response, with decreased activation of CD8+ cells and T regulatory cells (Tregs) in the spleen, and decreased CD4+ T cell numbers resulting in an altered CD4/CD8 T cell ratio in the CNS coupled with CNS disease. Our observations imply an essential role for STING within the interface between the innate and adaptive immune responses for effective immune programming in the control of WNV infection and CNS disease.

## Results

### STING is required for host defense in the CNS during peripheral WNV infection

**Fig 1:**
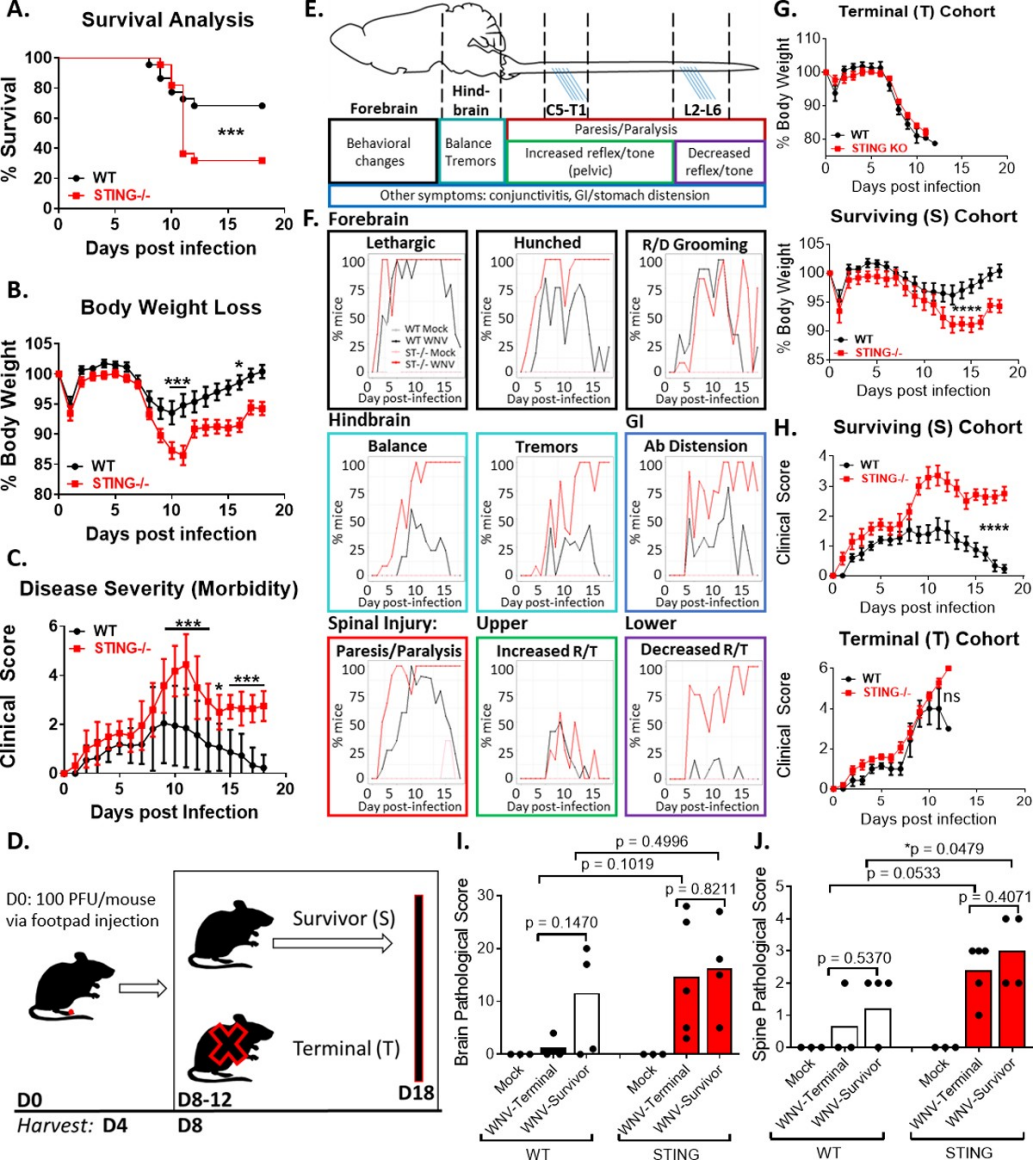
STING deficiency leads to increased morbidity and mortality during WNV infection in vivo. (A) Increased mortality in STING-/- mice. n = 22 per strain; Mantel-Cox analysis, p = 0.05*; p = 0.005**, p = 0.0005***. (B-C) Body weight loss (B) and clinical scores (C) are more pronounced in STING-/- mice, indicating increased morbidity during WNV infection. n = 22 per strain; two-way ANOVA, Bonferroni posttest; p = 0.05*; p = 0.005**, p = 0.0005***. (D) Schematic of infection and harvest time points. (E) Schematic of clinical signs and predicted pathology associated with WNV damage of the CNS. Anatomical model and clinical associations modified from previously described studies [85–89]. (F) Clinical signs observed during WNV infection of WT and STING-/- mice. R/D: ruffled/decreased; Ab: abdominal; R/T: reflex/tone. n = 10 per strain. (G) Body weight loss in (top) Terminal (T) vs (bottom) Survivor (S) cohorts. n = 22 per strain; two-way ANOVA, Bonferroni posttest; p = 0.05*; p = 0.005**, p = 0.0005***. (H) Clinical score analysis in (top) Terminal (T) vs (bottom) Survivor (S) populations. n = 22 per strain; two-way ANOVA, Bonferroni posttest; p = 0.05*; p = 0.005**, p = 0.0005***. (I-J) Pathological damage observed in the brains (I) spinal cord (J) by H&E staining in WT and STING-/- Terminal (T) and Survivor (S) mice. n = 3–9 per condition; students t-test (unpaired); p = 0.05*; p = 0.005**, p = 0.0005***.

Previous studies demonstrated that mice defective in STING signaling experienced increased mortality during WNV infection, yet the linkage of STING to immune response programming for defense against WNV has not been defined [46]. Using genetically knocked-out *Tmem173* (STING-/-) mice [67], we first performed a survival analysis to confirm the role of STING in host survival during WNV infection (Fig 1A). C57B/6J (B6, WT) and STING-/- mice were infected through subcutaneous virus challenge via foot-pad injection and monitored for 18 days post infection (dpi). Mice were scored daily for morbidity, marked as loss in body weight (Fig 1B) and overall increased clinical score (Fig 1C). Consistently, between 8–12 dpi, mice either met euthanasia criteria (Terminal; T) or went on to survive (Survivors; S) through 18 dpi (study end-point) (Fig 1D). Using this model, we confirmed the occurrence of increased susceptibility to WNV infection in the complete absence of STING (Figs 1A and S1A), similar to what was previously described in STING^gt/gt^ mice [46]. We also observed significantly increased clinical severity scores in the STING-/- mice that persisted until the study-endpoint, when WT mice had returned to a base-line clinical score (Fig 1B and 1C). Additionally, we monitored mice daily for the duration of the experiment until they either met euthanasia criteria or at the study end-point, day 18 post infection. Results from each mouse were analyzed to determine if there were differences in clinical signs between WT and STING-/- mice. Notably, STING-/- mice displayed increased neurological signs of disease, characterized by loss of balance, reduced muscle tone and reflexes predominantly in the pelvic limbs and increased paresis and paralysis, implicating more severe damage to the hind-brain and spinal cord (Fig 1E and 1F). In order to determine if there was a survivor bias in the clinical data, we retrospectively stratified the data into cohorts of mice that met euthanasia criteria (Terminal; T) or ones that survived until day 18 post-infection (Survivors; S), the pre-determined study end-point (Fig 1D, 1G and 1H). By doing so, we found that significant differences in body weight loss and clinical scores between WT and STING-/- mice were only observed in the Survivor cohort and not in the Terminal cohort. While there is an essential role for STING in host survival during acute infection (Figs 1A and S1A), these data implicate an additional prolonged requirement for STING in both prevention and recovery from neurological pathology. When we examined CNS pathology, we found that in both WT and STING-/- mice, pathological scores were significantly increased in the spines of the Survivor cohort, with a trend toward increased scores in the brains and spines of the Terminal cohort (Fig 1D, 1I and 1J). Intriguingly, while STING-/- Terminal mice displayed increased CNS pathology, WT mice that met Terminal criteria had unexpectedly low clinical scores, suggesting that they met euthanasia criteria for reasons independent of severe encephalitis. During necropsy, we observed that the gastro-intestinal (GI) tract of Terminal mice exhibited gross distension or other aberrant phenotypes including stool compaction, disintegration and in some cases severe reduction in size or collapse of the GI tract (S1B Fig). Pathologic analysis confirmed that Terminal mice display increased GI pathology that included microbiome overgrowth and neuronal degeneration and loss in the myenteric ganglia, particularly in STING-/- (S1C and S1D Fig). Previous studies have indicated that GI manifestations during WNV infections exist in both mice and humans, and are positively correlated to increased neurotropism and mortality [15–17, 22]. This outcome may imply that WT mice are meeting euthanasia criteria following WNV infection due to severe GI disease rather than severe CNS involvement as previously thought. Further, these results demonstrate that STING plays a systemic role in host defense against WNV, with increased frequency of mortality and pathology occurring in the CNS and GI tract in STING-/ mice. Together, these results show an essential role for STING in host survival and neuropathological defense in the CNS during WNV infection.

### Protective role of STING is not initiated in neurons or the CNS

**Fig 2:**
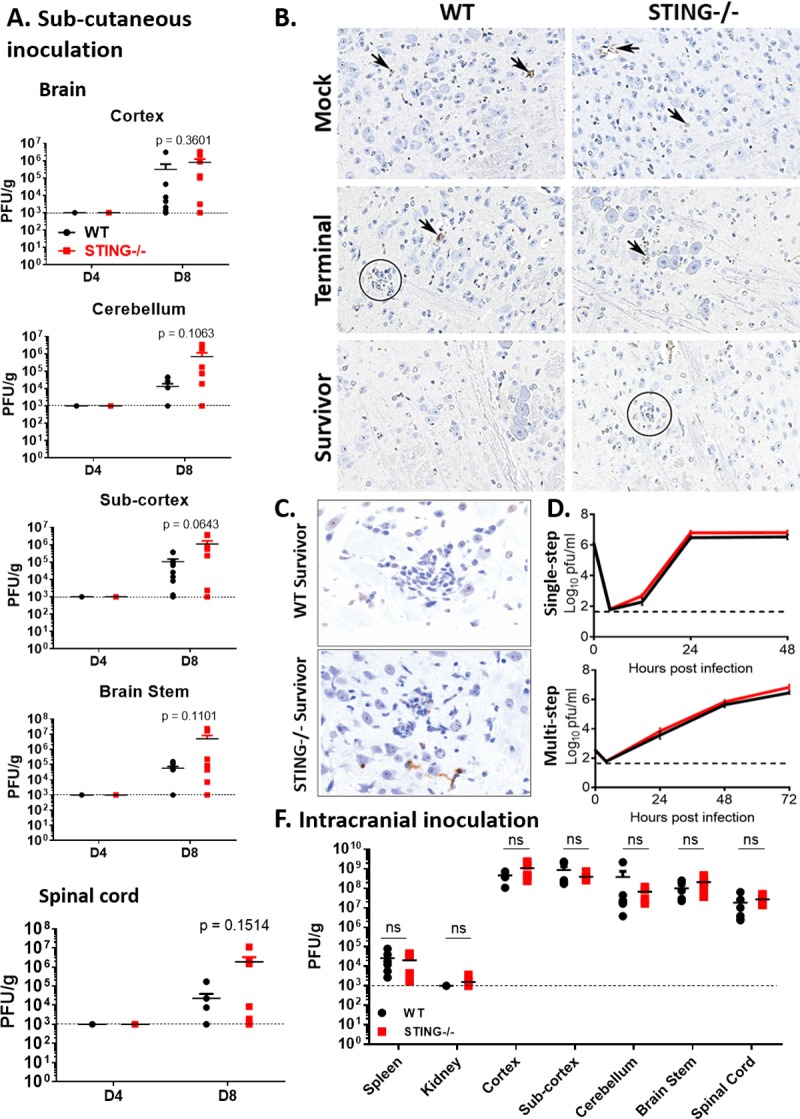
STING is not required for viral control of WNV in neurons *in vitro* or in the CNS during intracranial infection *in vivo*. (A) WNV viral load in macro-dissected brain sections (cortex, sub-cortex, cerebellum and brain-stem) and spinal cord of WT and STING-/- infected mice, D4 and D8 post infection. n = 6–10 per strain per time-point. Graphed as stacking points. Limit of detection indicated by dashed line. Unpaired students t-test; p = 0.05*; p = 0.005**. (B) WNV IHC in the brains of mock infected and WNV infected WT and STING-/- Terminal and Survivor cohort. Mock tissues are unremarkable with non-specific staining of capillaries (arrows). Terminal mice have punctate staining near foci of gliosis (WT, circle) or neuronal degeneration (WT and STING-/-, arrows). No discernable specific signal for WNV antigen was observed in either WT or STING-/- Survivors, despite observable gliosis in STING-/- (circle). All panels, original magnification 200X. (C) TUNEL IHC stains of representative WT and STING-/- Survivor (18 dpi) mice. Brown stain indicates neuronal death. (D). Single and multistep virologic analysis of primary cortical neurons from WT and STING-/- mice. Pooled samples of 3 embryos per genotype. (E) Titer in mice infected with WNV via intracranial inoculation D4 pi. n = 6 WT and n = 5–6 STING-/-. Students t-test, p = 0.05*; p = 0.005**.

To determine if STING is required for viral control in the CNS, we challenged mice with WNV via footpad injection and examined tissue viral load at 4 dpi (peak of peripheral viremia) and 8 dpi (peak of detectible virus in the CNS) (Fig 2A). Viral titer of macrodissected brains and extracted spinal cords were examined by plaque assay individually for each mouse in the cohort (Fig 2A). As expected, virus was not detected at 4 dpi in the CNS but by 8 dpi virus was clearly detected in different CNS regions. Virus was not consistently found in the CNS of all mice nor in every tissue examined. There was however, a consistent trend toward increased numbers of infected mice with detectible virus in the CNS as well as increased viral titers in the CNS of STING-/- mice compared to WT. To determine if there was detectible virus in the brains of Terminal vs Survivor mice, tissues from retrospectively sorted mice utilized for pathological analysis (Fig 1I and 1J) were immunostained for the presence of WNV antigen (Fig 2B). WNV foci were found in the brains of WT and STING-/- Terminal mice but were not apparent in WT or STING-/- Survivors, suggesting that either the virus had cleared or that surviving mice did not have CNS infection. Neuronal death was assessed by TUNEL stain in both WT and STING-/ Survivors. Here we observed enhanced neuronal apoptotic death in the STING-/- cohort, suggesting STING may have a direct or indirect role in neuronal defense in the CNS (Fig 2C). In order to determine if STING is required for neuronal defense against WNV, primary cortical neurons were isolated and cultured, followed by infection with WNV to determine viral growth kinetics under conditions of single and multi-step growth (Fig 2D). Surprisingly, no difference was detected between in WNV replication in WT and STING-/- primary cortical neurons (Fig 2D). To determine if the actions of STING might be restricted to the CNS for WNV protection, we performed an intracranial virus inoculation bypassing the role of the peripheral immune response and physical barriers such as the blood-brain barrier to directly infect the brain with WNV (Fig 2E). At 4 dpi, there was no difference in CNS viral load found in WT vs STING-/- mice nor was viral load different between STING-/- and WT mice. Taken together, our observations imply that the role of STING is not limited to mediating viral control in the CNS. It is possible that STING is therefore required in the development of a protective immune response in the periphery such that in the absence of STING the immune response is aberrantly programmed, leading to CNS immunopathology.

### Innate immune response to WNV is intact in STING-/- mice

**Fig 3:**
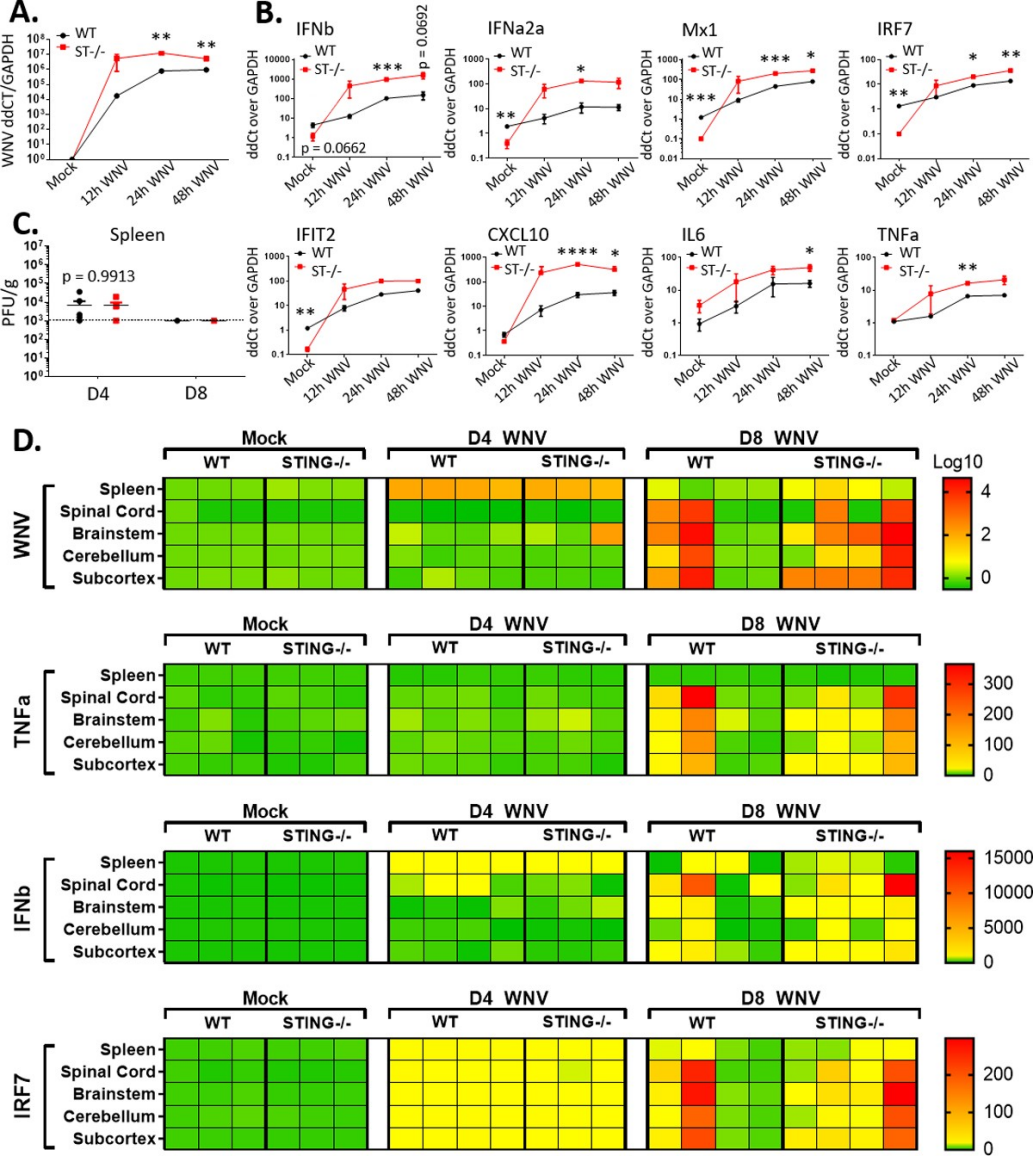
STING deficiency leads to increased innate immune signaling during WNV infection. (A-B) (A) WNV detection in BMDM by RT-qPCR, (B) innate immune response gene expression in WNV-infected BMDM over an infection time course. Bone marrow was harvested and differentiated into BMDM with mMCSF for 7 days. Cells were infected and harvested at the indicated time-points. Mock infected cells harvested at 12hpi. n = 3 infectious replicates. Results were reproducibly significant in multiple studies. Calculated as linear fold change over WT Mock. Unpaired students t-test; p = 0.05*; p = 0.005**, p = 0.0005***. (C) Splenic WNV titers at D4 and D8 post infection (PFU/g detected by plaque assay). n = 3–4. Graphed as stacking points. Limit of detection indicated by dashed line. Unpaired students t-test; p = 0.05*. (D) *In vivo* innate immune profile in splenic and CNS tissues. RT-qPCR detection of innate immune genes in the spleen, spinal cord and brain regions (brain stem, cerebellum, sub-cortex). Columns indicate individual mice; rows the different tissues. Calculated as log (WNV) or linear (immune) fold change over GAPDH in WT Mock. Dark red indicates values outside of (above) set scale.

Given that STING deficiency was associated with enhanced mortality (see Fig 1) without a significant increase in CNS viral burden (Fig 2), we considered that STING deficiency could result in defective antiviral innate immune signaling and lead to loss of viral control in the periphery, thereby leading to enhanced morbidity and mortality. We first tested the role of STING in BMDMs, as macrophages are a tropic cell and key modulator of peripheral viral control during WNV infection (Fig 3A) [19]. As expected, WNV levels were significantly increased by 24 and 48 hours post inoculation (hpi). Unexpectedly however, STING-/- BMDM had increased innate immune and inflammatory gene expression, including enhanced level of type I IFN expression during WNV infection (Fig 3B). We then examined the spleens of infected mice to determine if there was an overall loss of viral control manifested as increased viral load over WT. As expected, virus was detected at 4 dpi in both WT and STING-/-. Surprisingly however, there was no difference in 4 dpi viral titers between WT and STING-/-, nor was there a sustained virologic response in STING-/- mice (Fig 3C). These data indicate that peripheral loss of viral control does not occur in the absence of STING (Fig 3C). Similarly, viral RNA was detected equally in spleens of infected WT and STING-/- mice at 4 dpi, but the virus was largely cleared from the spleen by 8 dpi (Fig 3D). In the CNS however, we observed a trend toward increased viral RNA and innate immune gene expression at 8 dpi in WNV-infected STING-/- mice, similar to that observed in BMDM (Fig 3A and 3D). These data were unexpected as we initially predicted that STING deficiency would reduce innate immune activation based on the known role of STING signaling in IFN induction. These data demonstrate that innate immune activation and the inflammatory response are exacerbated in both *in vitro* and *in vivo* STING deficient models, possibly culminating in enhanced immunopathology in STING-/- mice.

### STING has a non-canonical role in host defense against WNV infection

**Fig 4:**
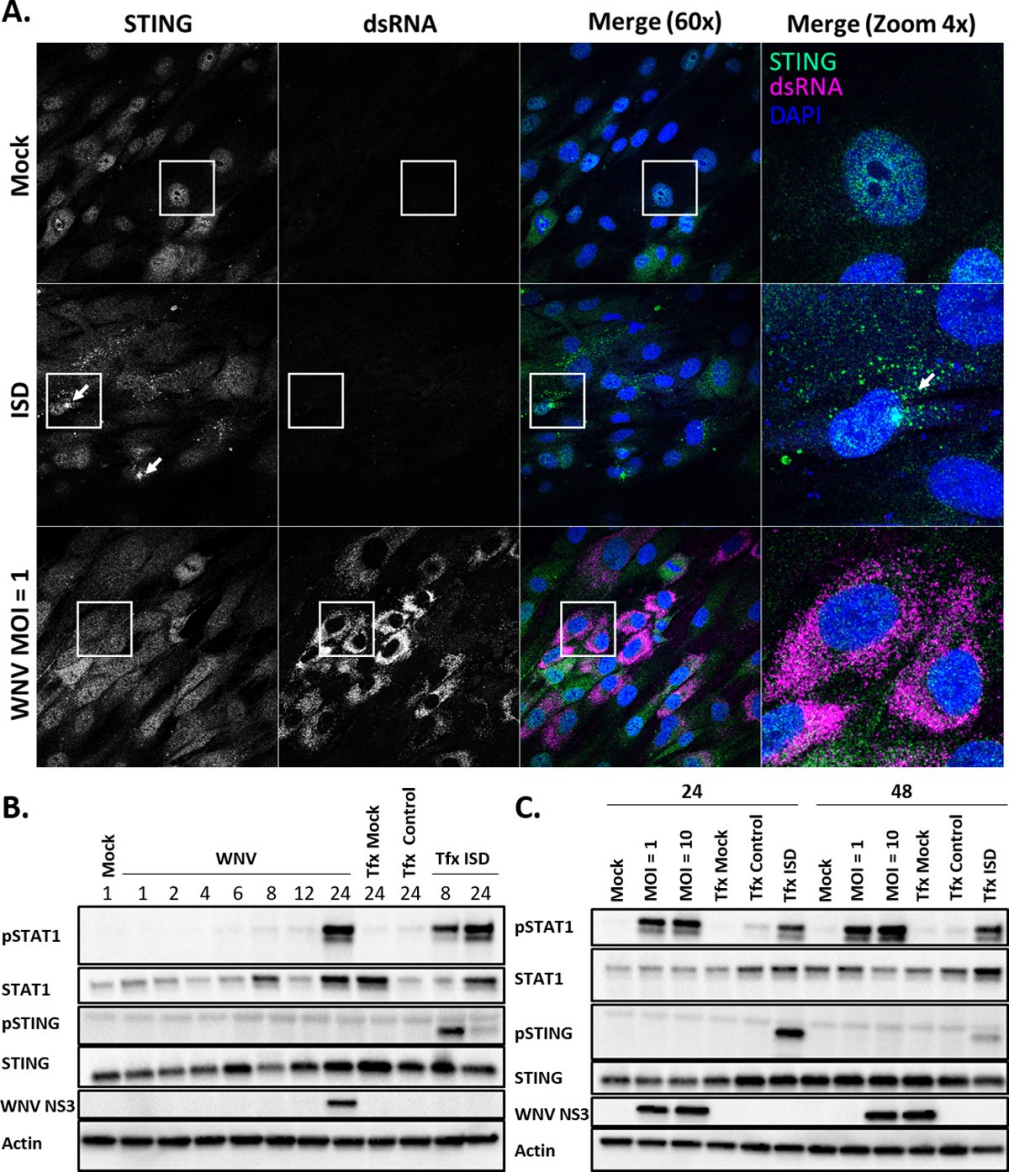
STING is not activated during WNV infection. (A) Immunofluorescence STING re-localization assay to assess STING activation. HFF cells were treated with PBS (Mock), transfected with ctDNA (ISD) for 3hr infected with WNV for 24h (MOI = 1). Cells were stained for endogenous STING and dsRNA. The data are representative for two independent experiments. (B-C) Western blot to detect activation of endogenous STING during WNV infection. (B) HFF were infected with WNV MOI = 1 and harvested at the indicated times post inoculation (hpi). (C) HFF cells were infected with WNV (MOI = 1, MOI = 10) and lysed at 24 and 48 hpi. In parallel, HFFs were transfected with ctDNA and harvested at the indicated times (B-C). The data are representative of three replicate experiments.

The canonical STING sensing pathway is dependent on upstream recognition of DNA danger- or pathogen-associated molecular patterns (DAMP, PAMP) such as DNA viruses, cell-free or mitochondrial DNA, by cyclic GMP-AMP synthase (cGAS). In mammals, cGAS binding to dsDNA activates its synthase activity to produce a cyclic di-nucleotide, cGAMP (cyclic guanosine monophosphate-adenosine monophosphate), which binds to STING, initiating downstream activation of STING by phosphorylation, STING relocalization from diffuse cytosolic to punctate pattern, and subsequent induction of innate immune signaling and IFN production [47, 48, 53, 68, 69]. During RNA virus infections however, the role for STING defense has not been well-characterized. To evaluate the activation of STING during WNV infection, we utilized a recently described telomerase reverse transcriptase human foreskin fibroblasts (HFF) model to assess activation of endogenous STING by phosphorylation and relocalization from the cytoplasm to the perinuclear space during WNV infection [70]. Transfection of interferon-stimulated DNA (ISD; calf-thymus DNA) into HFFs initiated re-localization of STING as previously reported by 3hpi [48, 70]. Intriguingly however, STING was not relocalized in WNV infected cells (Fig 4A). It is possible that the kinetics of STING activation are different from ISD activation of STING as compared to WNV infection, so we performed a time course experiment to detect STING activation by phosphorylation status [71], assessing a range of 1–24 hpi at MOI = 1 (Fig 4B). Similar to what was observed by IFA, STING phosphorylation was not observed at any time point during WNV infection, although phosphorylated STAT1 and WNV protein was detected at 24 hpi, suggesting virus replication and innate immune signaling were occurring normally (Fig 4B). To determine if activation was dependent on viral load, we infected HFF with a MOI = 1 and MOI = 10 of WNV, but also observed no STING activation as measured by phosphorylation (Fig 4C). These data suggest that STING is not canonically activated during WNV infection in HFF cultures and reveals a potential non-canonical role for STING in host defense during infection with WNV.

### STING affects development of the adaptive immune response to WNV

In order to determine if there was a systemic change in the innate immune profile in STING-/- mice, we examined the cytokine and chemokine profile in the serum of WT and STING-/- mice at the peak of peripheral viremia (4 dpi) and CNS viral burden (8 dpi). We found that mock infected STING-/- mice had an increased basal production of multiple cytokines and chemokines at 4 dpi. We also observed significant increases in IL33, IL4, IL6, IL15, MCSF, Gro-alpha, while at 4 dpi IP-10 (CXCL10) was decreased in STING-/- compared to WT mice (S2 Fig). While these cytokines have multiple roles in immune modulation, a common role among them is in activation and recruitment of T cells. These data suggest that STING is required for regulation of immune cytokine and chemokines that program immune cell trafficking and actions during WNV, as has been shown for STING in cancer immunity and autoimmune signaling [53].

**Fig 5:**
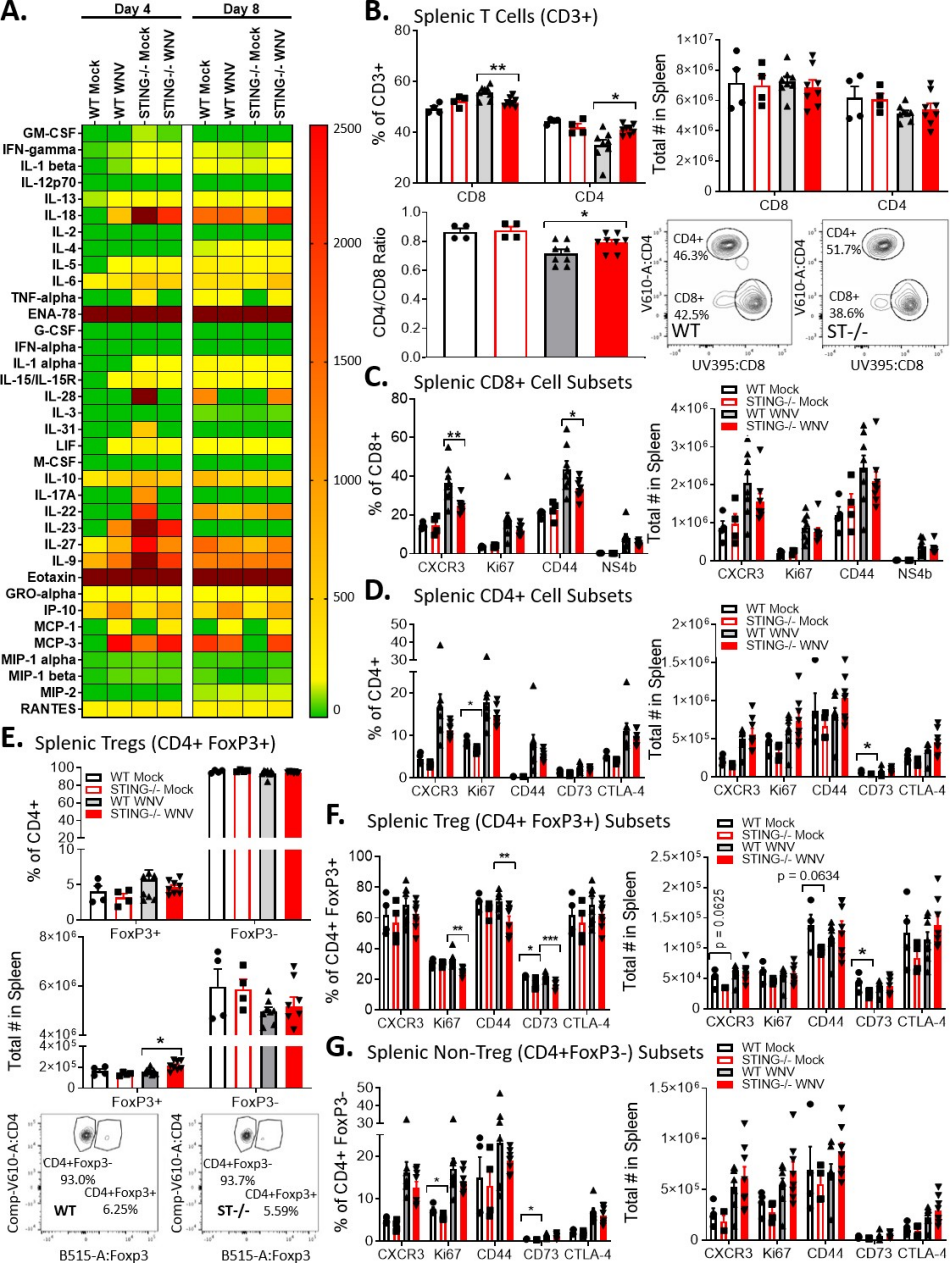
STING is required to program the adaptive immune response during WNV infection. (A) Luminex analysis of cytokines and chemokines from serum in mock (PBS) and WNV infected mice at D4 and D8 post infection. n = 3–8 per condition. Dark red indicates values outside of (above) set scale. (B) CD4+ and CD8+ T cell frequency and absolute number in D8 post infection spleens (top). Flow schematic (bottom left) and CD4/CD8 ratio (bottom right). n = 4–8 per condition. Unpaired students t-test; p = 0.05*; p = 0.005*; p = 0.0005***). (C) Characterization of D8 pi splenic CD8+ T cell sub-populations (CXCR3+, Ki67+, CD44+ and NS4b+). Left: frequency; Right: Absolute number. n = 4–8 per condition. Unpaired students t-test; p = 0.05*; p = 0.005*; p = 0.0005***). (D) Characterization of D8 pi splenic CD4+ T cell sub-populations (CXCR3+, Ki67+, CD44+, CD73+ and CTLA-4+). Left: frequency; Right: Absolute number. n = 4–8 per condition. Unpaired students t-test; p = 0.05*; p = 0.005*; p = 0.0005***). (E) Splenic FoxP3- and FoxP3+ CD4+ T cells. Top: frequency; Middle; absolute number; Bottom: gating scheme. n = 4–8 per condition. Unpaired students t-test; p = 0.05*; p = 0.005*; p = 0.0005***). (F-G) Analysis of FoxP3+ (F) and FoxP3- (G) subpopulations (CXCR3+, Ki67+, CD44+, CD73+ and CTLA-4+). Left: frequency; Right: Absolute number. n = 4–8 per condition. Unpaired students t-test; p = 0.05*; p = 0.005*; p = 0.0005***). B-F: WT, wild type; ST-/-, STING-/-.

To determine if STING is required for proper programming of the T cell response during WNV infection, we examined splenic T cells from WT and STING-/- mice at 8 dpi, a time point when the adaptive immune response is established in WT mice [24]. We observed a reduction in the frequency of CD8+ T cells, along with a trend toward decreased numbers of T cells in the spleens of STING-/- mice compared to WT during WNV infection (Fig 5B). Additionally, within the CD8+ T cell subset (Fig 5C), there was a significant decrease in frequency of activated (CD44+) and CXCR3+ T cells, and we observed a consistent trend of decrease in the frequency of WNV-specific CD8+ T cells in the spleens of STING-/- mice compared to WT, suggesting that STING is required for optimal anti-WNV CD8+ T cell responses. We also observed a significant increase in the frequency of CD4+ T cells in STING-/- mice (Fig 5B), with a corresponding trend toward increased absolute cell numbers. While we observed a trend toward differences in the absolute number of most cell populations examined between WT and STING-/- mice, we found that significant differences most typically occurred in cell frequencies, suggesting that the balance of T cells subsets may be skewed in the absence of STING. In particular, we found skewing within the T regulatory cell (FoxP3+) populations (Fig 5E–5G), with significant deficits in Ki67+, CD44+ and CD73+ Tregs, CD44 and CD73+ Tregs. These data suggest that STING is required for modulating T cell responses and T cell frequencies during WNV infection that lead to a protective rather than pathogenic outcome.

### STING is required for development of a protective adaptive immune response to WNV in the CNS

**Fig 6:**
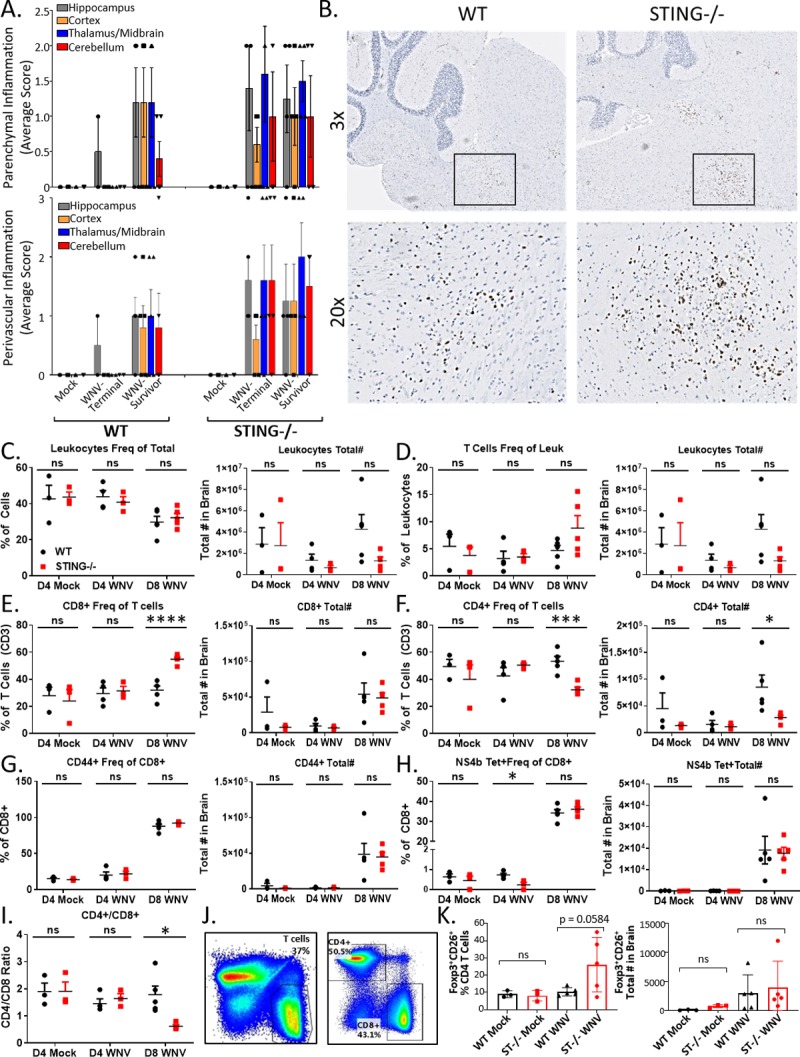
STING-/- have a defective adaptive immune response in the CNS during WNV infection. (A) Cellular infiltrate in the brain in Terminal and Survivor mice by pathological review of H&E. n = 3–5. (B) CD3 IHC of WNV infected brains. Boxed regions, higher magnification, lower panel. T cells stain brown; hematoxylin counterstain. (C-H) Flow analysis of brain lymphocytes. Left: frequency; Right: Absolute number. N = 3–5 per condition. Unpaired students t-test; p = 0.05*; p = 0.005*; p = 0.0005***). (I) CD4/CD8 ratio in the brain. (J) Flow gating scheme. (K) FoxP3+CD25+CD4 T cells in brains. Left: frequency; Right: Absolute number. N = 3–5 per condition. Unpaired students t-test; p = 0.05*; p = 0.005*; p = 0.0005***).

Because of the heightened innate immune profile and aberrant programming of the T cell responses in spleens of STING-/- mice, we examined the CNS-specific T cell profile across mouse lines. Histological analyses revealed trends toward increases in CNS immune cellularity, both in the form of perivascular and parenchymal mononuclear infiltrate, suggesting the CNS pathology may be immune-mediated (Fig 6A). We then performed a CD3 IHC stain in the brains of Survivors, we found increased clusters of CD3 infiltrate in the hind and mid-brain regions (Fig 6B) co-localized with robust lesions. In serial slices of the same tissues, we did not observe WNV staining by IHC in STING-/- Survivors (Fig 2), however we did observe continued gliosis, suggesting that a potential immunopathology may occur in the brain of STING-/- mice infected with WNV. Previous studies indicated that cellular infiltrate in the brain is predominantly comprised of CD3+ T cells during WNV infection [72]. Therefore, we characterized T cell responses of WT and STING-/- mice in the CNS on 4 dpi to examine baseline differences at 8 dpi when WNV and leukocytes are both present in the CNS (Fig 6J). Lymphocyte and T cell responses in both mock and WNV-infected mice were comparable at 4 dpi, indicating that there was no gross difference in the CNS between WT and STING-/- mice (Fig 6C and 6D). By 8 dpi however, we found statistically significant decreases in the frequency and numbers of CD4+ T cells in STING -/- mice (Fig 6F). Although there was no difference in the total numbers of CD8+ T cells, there was a statistically significant increase in the frequency of CD8+ T cells in the CNS of STING-/- mice, likely due to overall trend of decreased numbers of lymphocytes in the brain (Fig 6C–6E). By 8 dpi, these changes resulted in a significantly decreased CD4/CD8 ratio of T cells, indicating an imbalanced T cell response to WNV in the CNS of STING-/- mice (Fig 6I). Of cells that made it to the brain by 8 dpi, no differences were found in the absolute number of activated (CD44+) or WNV-specific (NS4b Tetramer+) CD8+ T cells (Fig 6G and 6H), FoxP3+CD25+CD4+ T cells (Fig 6K) in the brain. These data suggest that STING is not essential for recruitment of WNV-specific cytotoxic T cells in the CNS, however it may be required for balancing the cytotoxic vs immunosuppressive adaptive response. Furthermore, it is also possible that the enhanced recruitment of cells to the CNS is in response to damage caused by the virus, aberrant immune signaling, or both. This outcome would suggest that STING plays an essential role in modulating the balance between immunopathogenic and immunoprotective response in the CNS during WNV infection.

### Neuropathology and viral loads increase in the CNS of STING deficient mice

**Fig 7:**
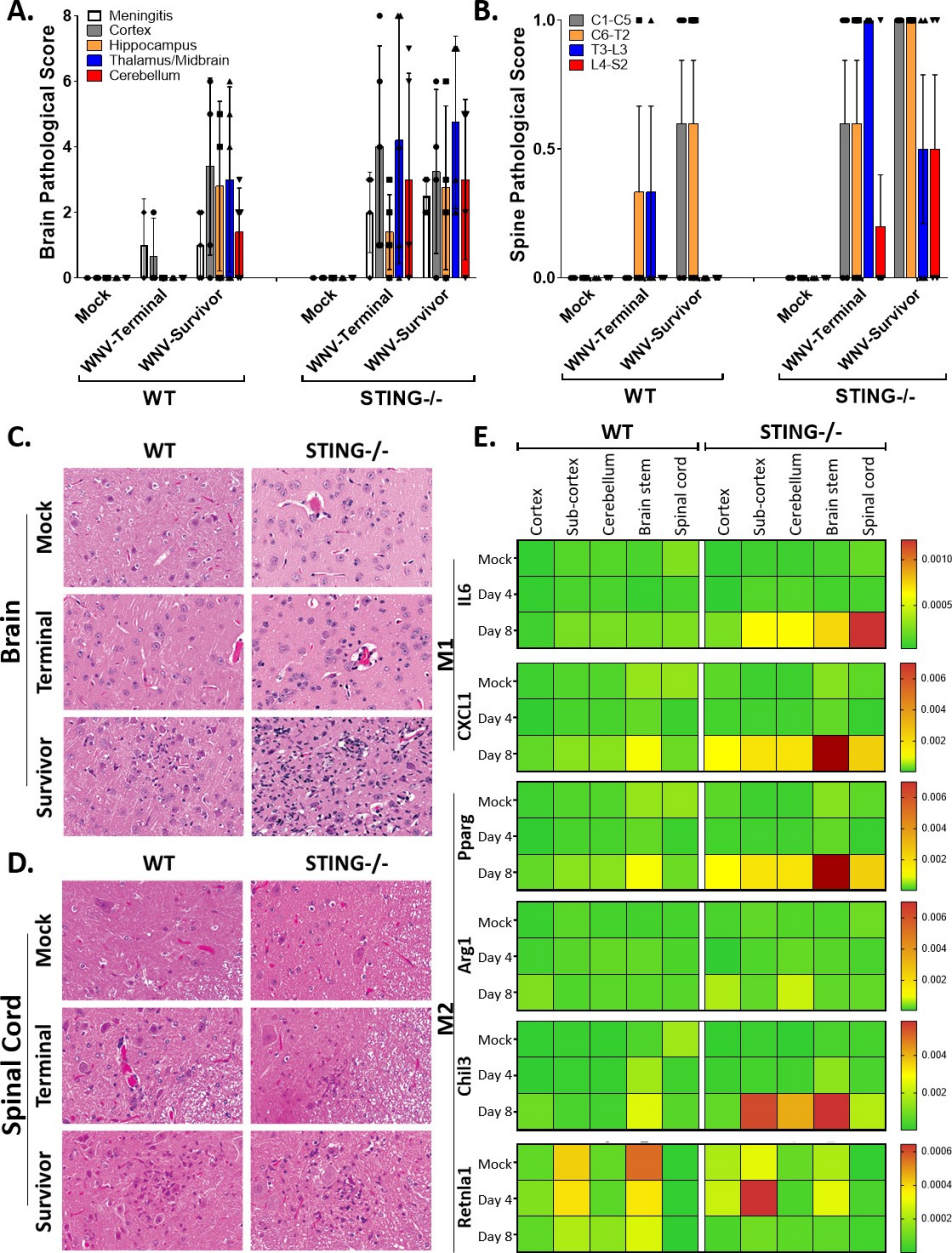
STING is essential in limiting neuropathology and WNV in the CNS. (A-B) Regional analysis of pathology in WT and STING-/- brains (A) and spinal cords (B) in Terminal (mice meeting euthanasia criteria) and Surviving (harvested at study endpoint) mice. n = 3–5 per condition. (C-D) Representative H&E of pathological lesions and cellular infiltration in Brain (C) and Spinal Cord (D). (C) Mock tissues are unremarkable. Terminal mice have minimal lesions, observable in STING-/- as perivascular mononuclear cells and minimal gliosis. For both genotypes, Survivor mice have readable observable neuropathology which is severe in the STING-/- mice with intense gliosis, neuronal degeneration and death and perivascular cuffing with mononuclear cells. In the WT Survivors, there is a mild focus of gliosis and neuronal degeneration. All panels, original magnification 200X. (D) Mock tissues are unremarkable. Terminal mice have minimal lesions, observable as perivascular mononuclear cells. For both genotypes, Survivor mice have readable observable infiltration of mononuclear cells and neuronal degeneration. All panels, original magnification 200X. (E) M1 and M2 gene expression analysis by RT-qPCR. Calculated as relative fold change. n = 3–5 per condition.

The increase in clinical disease and pathological damage observed in the STING-/- versus WT mice, particularly in Survivors, could be due to an aberrant immune response resulting in CNS damage after initial viral insult. We found that CNS pathology in WT mice is largely restricted to the cortex and meninges, while STING-/- mice display increased pathology in the cerebellum and hind/mid brain regions in addition to the cortex and meninges (Fig 7A and 7C). These data correlate with the increased CD3 staining observed by IHC in STING-/- mice (Fig 6B), also noted as the same brain regions where WNV is often detected by IHC (Fig 2B). These observations suggest that STING plays a role in directing or maintaining the T cell response to specific loci within the CNS or that initial viral infection led to increased recruitment of a localized adaptive immune response that resulted in immunopathology. Furthermore, pathology in the spine was more diffuse, suggesting that STING has a widespread protective role in the CNS during WNV infection (Fig 7B and 7D). These observations led us to investigate if there was a localized polarization of microglia or infiltrating macrophages in CNS regions toward an M1 or M2 phenotype (Fig 7E). Microglia have the highest levels of STING (*Tmem173*) expression observed in any cell within the adult mouse [73, 74] and it is possible that in the absence of STING, microglia are aberrantly polarized, enhancing immune-mediated pathology. To examine this possibility, we assessed the expression of M1 (CXC1 and IL6) and M2 (Pparg, Arg1, Chil3 and Retnla 1) associated genes by RT-qPCR in different regions of the CNS. In WT mice, we found that CXCl1 (marking an M1 phenotype) was present in the brain stem by day 8 post infection, and Retnla 1 expression (marking an M2 phenotype) occurred in both the mock and 4 dpi tissues within the brain stem and sub-cortex (containing the thalamus) regions of the brain (Fig 7E). This profile suggests that CNS homeostasis includes a localized M2 phenotype that is induced to a M1 phenotype in WT mice following WNV infection. In STING-/- mice however, we found a widespread increase in the M1 response gene expression (marked by CXCL1 and IL6) with the highest expression observed in the brain stem and spinal cord. Simultaneously, there was also a corresponding increase in Pparg and Chil3 (marking the M2 phenotype), with no clear difference in Arg1 expression and an overall trend toward decreased expression of Retnla. These observations reveal a widespread increase in both M1 associated genes, with altered regulation of the M2 associated genes in STING-/- mice, potentially resulting in aberrant balance of the M1 and M2 polarization in the CNS. To determine where in the CNS STING is actually localized and if this tissue localization overlaps with the location of the cellular infiltrate noted histopathologically or with expression of innate immune genes, we utilized the Allen Brain Institute database to search for STING (*Tmem173*) localization in the mouse brain [75]. Within the brain, STING expression is found within the olfactory bulb, thalamus/midbrain, brainstem and cerebellum, as well as low levels throughout the cortex, overlapping areas that are affected most severely by WNV infection (S3) [14, 75]. These regions of brain affected correlate with the clinical signs we observed including loss of balance, tremors, and loss of motor function (Figs 1E and 7C–7E). Furthermore, these areas of STING expression overlap with the brain regions where altered regulation of M1 or M2 gene expression were most readily observed, implicating a role for STING in polarization of either or both microglia and macrophages in the CNS. Cumulatively, these data suggest that STING has an essential role in maintaining immune response homeostasis and immune programming in initial defense against WNV infection. Without STING, immunopathology occurs, leading to exacerbated CNS disease and clinical sequelae.

## Discussion

Recent years have seen a marked increase in the global health threat presented by emerging and re-emerging encephalitic viruses, particularly those with increased neurotropism and neuropathology such as WNV [1, 3, 10, 76, 77]. Previous studies indicated an important role for STING in host survival during WNV infection [46], however it is unclear what role STING plays in conferring host defense against RNA viruses [52, 54]. Here, we demonstrate that STING is essential to prevent host morbidity and mortality during WNV infection where it plays a role in immune homeostasis and programming. However, STING is not canonically activated *in vitro* upon infection with WNV, revealing a novel function for STING during infection with RNA viruses. Furthermore, we show that STING is essential for host neuropathological defense against WNV through regulation of the innate-adaptive immune interface *in vivo*.

We found that STING deficient mice exhibit increased mortality and morbidity including increased and sustained neurological clinical signs, particularly in mice that survive infection (Fig 1). These data were corroborated by pathological analysis, which also revealed distinct differences in CNS pathology. Intriguingly, there seems to be a stratification in clinical and pathological findings between the STING-/- mice that meet euthanasia criteria and those that go on to survive. Survivorship bias has been previously reported in the WNV model, with these data further implicating this bias as a critical factor to consider when performing time course vs. end-point experiments [78]. Unexpectedly, these studies also revealed that there was minimal CNS pathology in WT mice that met euthanasia criteria. It is typically assumed that mice meeting euthanasia criteria do so because of neuroinvasion and subsequent encephalitis. Our data instead indicates that both WT and STING-/- Terminal mice have severe gross GI abnormalities, with corroborating abnormalities by histopathology, which may be the proximate cause of morbundity and meeting euthanasia criteria (S1). GI complications during WNV have been previously described, however further study is necessary to understand the implications of GI pathology on WNV induced morbidity and mortality [15–17, 79]. Recently it has been shown that during WNV infection causes delayed GI transit, dependent on infiltrating antiviral CD8+ T cells [80]. Furthermore, both in this model and in a lung model where STING exhibits a gain-of-function mutation, T cell-dependent chronic tissue damage occurs, supporting our findings that STING may play a broad and significant role in communicating between the innate and adaptive immune responses [80, 81]. Together, these data demonstrate an essential neuroprotective role for STING during WNV infection, potentially through a cellular mediated mechanism instead of the canonical interferon antiviral function typically attributed to STING.

WNV typically is cleared through development of an innate immune response and effective T cell immunity [19]. To prevent progression to neuroinvasion, both the innate and adaptive immune response are critical to control WNV viremia and prevent viral induced pathology [19–21, 24, 82, 83]. Because the known function of STING is to initiate a type I IFN response to both PAMPs and DAMPs, we anticipated that the type I IFN response would be diminished both *in vivo* and *in vitro* explaining the increased viral loads. Surprisingly, we actually observed an increased inflammatory and antiviral innate immune response in STING-/- mice in the CNS during WNV infection. This same increase in the cytokine-chemokine response was also observed in BMDM (Fig 3) and in serum of infected mice (Fig 5). These outcomes were highly unexpected as the most commonly described role for STING is known as initiating a type I IFN response [46–48, 53, 54]. In particular, STING was shown previously to facilitate the actions of the ELF4 transcription factor to promote type I IFN expression from WNV-infected cells wherein loss of STING associated with reduced IFN and ISG expression (49). While we observed significant increases in IFN and ISG expression in BMDM lacking STING, it is likely that STING imparts cell type-specific actions for regulation of innate immune signaling, similar to other pathogen recognition receptors that govern innate immune signaling against WNV, likely explaining this discrepancy between studies [19]. It is also important to note that our studies employed STING-/- mice produced through classical gene targeting approach [48] while the previous study used STING^gt/gt^ mutant mice produced from N-ethyl-N-Nitrosourea mutagenesis and encoding a T596A point mutation of STING [84], highlighting that genetic differences between mouse lines might impact findings. Importantly, both mouse lines exhibit increased susceptibility to lethal WNV infection, and together reveal expanded roles for STING in immune regulation during WNV infection.

Our data also suggest that STING has a role in controlling WNV replication and tropism, as we found increased viral loads in BMDM, as well as a trend toward increased viral load in the CNS, particularly in the hindbrain regions, but not in the spleens of infected mice lacking STING (Figs 2 and 3). The trend toward increased virus in the CNS of STING-/- mice could either suggest increased susceptibility of the virus in the CNS, delayed clearance of the virus after entering the CNS, or possibly a combination of the two. Variation observed within strains could be the result of harvesting mice at set time points instead of following them until a determination if they would survive or meet euthanasia criteria, highlighting the potential import of survivorship bias within this model. It does not appear that the requirement for STING in viral control is restricted to neurons or the CNS, as no difference was observed in the viral load of STING-/- primary cortical neurons or intracranial infection (Fig 2). This outcome suggests that while there is a peripheral requirement for STING in conferring CNS protection, it is not due to complete inhibition of viral control in the periphery. Intriguingly, base-line expression of type I IFN and ISGs were significantly reduced in STING-/- BMDM compared to WT, but not other inflammatory genes (Fig 3). It is possible that this reduction in baseline IFN allows WNV to establish an earlier and more robust infection, that is later controlled by the RIG-I dependent antiviral response [23, 34]. However, we favor that STING plays a role in innate immune homeostasis, as in its absence the control of the inflammatory response is lost (Figs 4 and 7), thus leading to immune-mediated pathology. This function for STING may explain why we had a trend but not significant increase in viral load in the CNS; it is possible that virus is able to establish a stronger infection in the CNS earlier on but is cleared through an exacerbated innate inflammatory and antiviral response in the absence of STING. Alternatively, it is possible that in the absence of STING clearance of the virus takes longer due to an ineffective immune response. Following either of these events subsequent T cell recruitment is likely, but in a manner that leads to enhanced immunopathology and lack of recovery from clinical illness.

In addition to its role in mounting a type I IFN response to PAMPs and DAMPs, recent studies demonstrated an essential role for STING in developing antitumor T cell responses [53]. These studies suggested that dead and dying cells are phagocytosed by dendritic cells, which requires STING to present antigen and produce a type I IFN signaling cascade that informs and develops the adaptive immune response. This outcome could also implicate a requirement for STING in microglial-dependent phagocytosis of dead and dying cells, with subsequent STING-dependent polarization and release of soluble factors that effectively recruit and maintain a protective cellular response in the CNS. Upon examining the CNS of infected mice, particularly in STING-/- with ongoing signs, we observed increases in mononuclear cellular infiltrate, implicating possible immunopathology. Previous studies have shown that there is an essential requirement for both CD8+ and CD4+FoxP3+ (Treg) T cells to control WNV and prevent immunopathology [42, 43, 72]. CD8+ T cells in particular are essential for WNV clearance, however without an adequate Treg response or appropriate balance of CD4+ and CD8+ T cells an uncontrolled cytotoxic T cell response could result in immune mediated pathology. Examining the programming of the adaptive immune response in spleens (Fig 5) we found that expression of Ki67, CD44 and CD73 in splenic FoxP3+CD4+ Tregs were impaired, implicating a role for STING in the proliferation, activation and suppressive potential of Tregs. Upon examining the brains of mice at baseline (4 dpi) and following infection (8 dpi), we observed no differences at baseline between WT and STING-/- mice, however the total CD4+ T cells and the CD4/CD8 ratio was significantly decreased in STING-/- mice, suggesting that there is a defective recruitment or maintenance of T cells in the brain (Fig 6). These data in combination with enhanced CNS pathology suggest that the cytotoxic effect of CD8+ T cells may not be controlled adequately in the absence of STING. It is also possible that increases in cellular response within the CNS recruit an enhanced protective cellular response as a result of viral damage or aberrant immune signaling. Consistent with this either of these options, we found that in STING-/- survivors there were large clusters of CD3+ cells (Fig 6) as well as other cellular infiltrate (Fig 7) in the same vicinity as we observed increased pathology and where STING is localized in the brain (Fig 7). Recently, a noncanonical STING-dependent signaling pathway was described where multiple cell types initiated an innate immune response following IL1b release in response to mitochondrial DNA release in the cytoplasm [70]. Furthermore, this STING-induced response to IL-1b was essential for the control of dengue virus infection, a flavivirus related to WNV [70] and that this response is linked with protection against WNV neurovirulence *in vivo* [70, 83]. Thus, it is intriguing to speculate that noncanonical STING activation in response to proinflammatory cytokine signaling serves to direct immune programming that protects against viral neuroinvasion and CNS pathology during WNV infection. In summary, our study reveals that that STING is required for immune response programming to restrict WNV infection and neuropathogenesis.

## Methods

### Ethics statement

All animal experiments were approved by the University of Washington Institutional Animal Care and Use Committee (IACUC) guidelines as per protocol #4158–05 and follow the recommendations in the Guide for the Care and Use of Laboratory Animals of the National Institutes of Health. Invasive infections and manipulations were performed under anesthesia and every effort was made to limit suffering.

### Animal sources

C57BL/6J (WT) and *Tmem173-/-* (STING-/-) mice were genotyped and bred under specific pathogen-free conditions in the animal facility at the University of Washington. STING-/- mice were gifted by the Stetson lab, who generated them as previously described [67] followed by speed congenics to bring them to a 99.4% C57BL/6J background. Additional C57BL/6J (WT) mice were purchased from Jackson Laboratories, Bar Harbor, ME. Both male and female mice, ages 8–11 weeks were represented in both the control and infected groups. Mice for primary cortical neurons (WT and STING-/-) were set up as timed breeders and embryos were harvested.

### Clinical scoring

Mice were monitored daily and assigned a clinical score to describe overall well-being and signs of hind-limb dysfunction (paresis). Clinical scores (CS) of (0) without clinical signs, or (1–6) dependent on severity of clinical signs presented. CS = 1: ruffled fur, lethargic; no paresis; CS = 2: very mild to mild paresis (in 1 or more hind limbs with minimal gait disturbance or limb-dysfunction); CS = 3: frank paresis involving at least one hind limb and/or eye conjunctivitis; CS = 4: severe paresis and/or paresis in both hind-limbs; CS = 5: true paralysis; CS = 6: moribund. Additionally, mice were observed daily for the presence or absence of various specific signs. Each mouse was scored as either exhibiting the clinical sign (YES = 1) or not, (NO = 0). Each sign was monitored through the duration of the experiment and the results were graphed as the average daily score/mouse. Results of clinical signs monitored represent the entire population until they reached euthanasia criteria, at which point the remaining mice continued to be scored until day 18 post infection or study end point. Clinical signs monitored daily include: Lethargy (L), Ruffled fur/decreased of grooming, Hunched, Paresis/Paralysis (any degree of severity), Tremors, Abdominal (Ab) distension/GI distress, Loss of Balance, Increased Reflex/Tone in limbs (fore and/or hind) and tail, Decreased Reflex/Tone in limbs and tail. The clinical scoring system incorporated signs based off of predicted involvement of different anatomical regions within the CNS and was created using modifications of various previously described scoring systems for experimental autoimmune encephalomyelitis [86–89]. Similar neuroanatomic regions were examined pathologically in an attempt to correlate clinical and neurological phenotype of disease.

### Survival analysis

Subcutaneously-infected mice were monitored for 18 days post infection (dpi). Euthanasia criteria was determined as a clinical score ≥ 5 for 2 or more consecutive days, or 20% loss in body weight. A clinical score of 6 (moribund) or respiratory distress resulted in immediate euthanasia. Mice meeting euthanasia criteria were identified as Terminal (T) and were euthanized by CO_2_ asphyxiation followed by cervical dislocation. Mice who did not meet euthanasia criteria were monitored until end point (18 dpi) were identified as Survivors (S). All remaining S mice were euthanized at the end of study (18 dpi) as described above.

### Pathology and pathological scoring

Mice used for morbidity and mortality analysis were necropsied when meeting euthanasia (T) criteria, or study end (S). After euthanasia by CO_2_, a complete necropsy was performed and tissues were collected and immersion fixed in 10% neutral buffered formalin [90]. The head was removed and skull cap lifted, leaving the brain within the skull cavity during fixation. The spine was fixed *in situ* in order to preserve the mesenteric ganglia. Histological preparation hematoxylin and eosin (H&E) and immunohistochemical (IHC) staining was performed by the UW Histology and Imaging Core (HIC) and the Vanderbilt University Medical Center Translational Pathology Shared Resource (TPSR). Primary pathological analysis was performed on the CNS (brain and spine) and gastrointestinal (GI) tract by a board-certified veterinary pathologist (PMT) (Supplemental methods Table 1). In the brain, the following changes were scored on a subjective 0–4 scale of increasing severity: perivascular inflammation, parenchymal inflammation, hemorrhage, neuronal necrosis, and meningitis. In the spinal cord the presence (1) or absence (0) of mononuclear inflammation was documented from 5 different sections of the spine (C1-C5, C6-T2, T3-L3, L4-S2, S3) for a maximum score of 5 per mouse. For the enteric nervous system (ENS), the degree of mononuclear cells present in the myenteric ganglia, extent to the changes and any secondary GI lesions such as dilation or mucosal change were scores on a on a subjective 0–4 scale of increasing severity. IHC staining of WNV (VRL W1015) and CD3+ T cells (MCA1477 AbD Serotec) were performed by the UW Histology Core.

**Table 1:** Histological scoring.

	Brain					ENS			Spine
Score	Perivascular inflammation: accumulation of inflammatory cells around vessels within Virchow-Robin's space	Parenchymal inflammation: inflammatory cells or gliosis with neuropil	Hemorrhage	Neuronal necrosis: including neurophagia	Meningitis	Mononuclear cells within the myenteric ganglia	Extent	Secondary lesions to GI	Mononuclear cells within the cord
**0**	None	None	None	None	None	None	None	None	None
**1**	Few cells <10	Few cells <10	Minimal	Minimal	Minimal thickening	Few cells <5	<25% affected	Minimal dilation, inflammation	Yes
**2**	Mild cells 11–20, slight expansion of space	Mild cells 11–20	Mild	Few necrotic bodies	Mild thickening, or minimal in multiple regions	Mild cells 6–20 with minimal neuronal damage	25–50% affected	Mild dilation, inflammation, or mucosal atrophy	
**3**	Moderate cells 21–30 and expansion up to 2X normal	Mild or moderate cells with parenchymal damage	Mild and multifocal	Multiple necrotic bodies or satellitosis	Mild or moderate expansion with superficial parenchymal damage	Mild cells 6–20 with evidence of necrosis or apoptosis	51–75% affected	Moderate dilation, inflammation, or mucosal atrophy, with necrosis or cell sloughing	
**4**	Perivascular cuffs up to 3X normal space	Marked cells and damage	Moderate	Marked numerous neuronophagic nodules	Marked expansion focally or moderate in multiple regions	Moderate cells >20 or no observable neurons	76–100% affected	Severe dilation, inflammation, or mucosal atrophy, with necrosis or cell sloughing, bacterial overgrowth	

### Cells

VeroWHO (European Collection of Authenticated Cell Cultures; ECACC) cells were cultured in Dulbecco’s modified Eagle medium (DMEM) supplemented with 10% FBS, 1mM sodium pyruvate, 2mM L-glutamine, antibiotic/antimycotic solution and non-essential amino acids (complete DMEM; cDMEM) and split using 0.25% Trypsin following PBS wash. HFF cells were kindly gifted from Stetson Lab and were grown in cDMEM. Cells were split using 0.05% trypsin following PBS wash. Bone marrow was collected from STING-/- and WT mice and frozen in 10% DMSO/90% FBS. To generate bone marrow derived macrophages (BMDM), bone marrow stocks were thawed, washed and resuspended in cDMEM containing [50 μM] BME and [40ng/mL] murine MCSF (mMCSF). Cells were cultured for 7 days in non-TC coated plates, then scraped, washed with PBS and seeded at 1E6 cells/well in 12-well TC coated plates with cDMEM+BME+mMCSF. Cells were infected or transfected the next day.

### Virus

WNV-TX biological isolates (2002) were utilized for *in vivo* work, while WNV-TX ic (infectious clone) stocks were utilized for cell culture (*in vitro*) studies. Working stocks were propagated in Vero-E6 (American Type Culture Collection; ATCC) and titered by standard plaque assay on VeroWHO and BHK21 (American Type Culture Collection; ATCC) cells as previously described [24]. Single-use aliquots from the same viral stock lot were prepared and utilized for all experiments described here.

### Subcutaneous infections

Age and sex-matched 8–11 week old mice were anesthetized by isofluorane and inoculated subcutaneously (s.c.) in the right rear footpad with 100 PFU WNV-TX 2002 (WNV-TX) diluted in 40 uL PBS, administered via 1mL insulin needle. Mice were monitored daily for clinical score and loss of body weight. Euthanasia criteria was determined as a clinical score ≥ 5 for 2 or more consecutive days, or 20% loss in body weight. A clinical score of 6 (moribund) or significant respiratory distress resulted in immediate euthanasia.

### Intracranial infection

Mice were anesthetized with ketamine/xylazine, the top of the head was cleaned with EtOH, and the mouse was then restrained manually on a solid surface. The site of injection was approximately halfway between the eye and ear, and just off the midline, in the medial posterior region of the top of the skull. The injection was done with a 29G needle using a Hamilton syringe into the cerebral cortex. Following infection, mice were monitored for revival from anesthesia and monitored daily for clinical score and loss of body weight. Euthanasia criteria was determined as a clinical score ≥ 5 for 2 or more consecutive days, or 20% loss in body weight. A clinical score of 6 (moribund) or significant respiratory distress resulted in immediate euthanasia.

### Viral quantification from tissues

To determine the viral load from *in vivo* tissue samples, mice were terminally anesthetized using ketamine/xylazine mixture followed by cardiac perfusion with 30–40 mL PBS. Kidney(s) and spleen were collected whole; brains were harvested and macrodissected into four anatomical regions, including the cerebellum, cortex, sub-cortex, and brainstem [91]; spinal cords were collected by perfusion with PBS. Tissues were harvested into 1 mL PBS on ice in Percelly’s tubes with ceramic beads. Following harvest, tissues were homogenized (Percellys 24) 5500/1x 20s/5 min and centrifuged at 4°C/5 min/10k rpm. Supernatant was collected and analyzed by plaque assay on Vero-WHO cells (0.5% agarose overlay, 3% Neutral red counter stain after five days post inoculation; plaques counted 10-15h post staining).

### WNV infections in tissue culture

Cells were inoculated with WNV in serum-free media and the inoculum left for 1hr rocking at 37°C. Inoculum was removed, cells washed 1x and media replaced with cDMEM. At the indicated time-points, supernatant was collected for virologic and cytokine analysis; cells were treated with RIPA buffer for WB analysis (or) with RLT for total cellular RNA isolation.

### Primary cortical neuron culture and infections

Primary cerebral cortical neuron cultures were generated from E15 WT and STING-/- embryos as previously described [92] and maintained in serum free Neurobasal-A medium (Life Technologies 21103–049) with B27 supplement (Gibco 17504–044). Neuron cultures were used for virologic experiments after 7 days *in vitro*. Cortical neuron cultures were infected at MOI 0.001 with WNV-TX [32]. Multistep growth curve experiments were performed as described [93] and quantified via plaque assay using BHK21-15 cells.

### Harvesting tissues for RT-qPCR analysis

Mice were euthanized in an isoflurane chamber followed by cardiac perfusion with 30–40 mL PBS. Tissues were harvested; right kidney and spleen were collected whole; brains were harvested and macrodissected into four anatomical regions, including the cerebellum, cortex, sub-cortex, and brainstem [91]; spinal cords were collected via PBS perfusion. Tissues were harvested into 1 mL RNALater and stored at 4°C for a minimum of 1 week to stabilize the RNA. Tissues were removed from RNALater solution and transferred to 1 mL TRIreagent in Percelly’s tubes with ceramic beads at RT. Following harvest, tissues were homogenized in a Percelly’s homogenizer (5500/1x20s/5 min) followed by centrifugation (4°C/10k rpm/5min). RNA isolated with the Ribopure kit from TRIreagent using per manufacturer’s instructions. cDNA was generated from 350 ng RNA using iSCRIPT kits with random primers per manufacturer’s instructions. Cellular and viral gene analysis was assessed by SYBR Green RT-qPCR using an ABI Viia7 and analyzed as the linear fold change (2^-dCT) over a housekeeping gene (GAPDH) from WT mock infected sample or mouse (Table 2).

**Table 2:** RT-qPCR Primers.

RT-qPCR Primers	Sequence	
mGAPDH	5’: CAACTACATGGTCTACATGTTC	3’: CTCGCTCCTGGAAGATG
WNV	F: TCA GCG ATC TCT CCA CCA AAG	R: GGG TCA GCA CGT TTG TCA TTG
mIFNb	F: GGAGATGACGGAGAAGATGC	R: CCCAGTGCTGGAGAAATTGT
mIFNa2a	Qiagen SABiosciences (PPM03543A)	
mIRF7	F: CCCATCTTCGACTTCAGCAC	R: TGTAGTGTGGTGACCCTTGC
mTNFa	TCCCAGGTTCTCTTCAAGGGA	R: GGTGAGGAGCACGTAGTCGG
mIL6	F: GTTCTCTGGGAAATCGTGGA	R: TGTACTCCAGGTAGCTATGG
mCXCL10	Qiagen SABiosciences (PPM02978E)	
mISG54 (IFIT2)	F: CTGGGGAAACTATGCTTGGGT	R: ACTCTCTCGTTTTGGTTCTTGG
mMX1	F: GACCATAGGGGTCTTGACCAA	R: AGACTTGCTCTTTCTGAAAAGCC

### RNA analysis from tissue culture

Cells were harvested in RLT and total cellular RNA isolated for RT-qPCR analysis using Qishredders and the Quiagen RNeasy kit per the manufacturer instructions. cDNA was generated from 100 ng total RNA using the iSCRIPT kit per manufacturer instructions using their provided oligo(dT) and random primers. Cellular and viral genes were analyzed by SYBR Green RT-qPCR using an ABI ViiA7. Primers for BMDM experiments described above.

### Protein analysis from tissue culture

Protein extracts from cells were prepared in RIPA buffer. 7–15 ng protein lysate was analyzed by 4–20% gradient SDS-polyacrylamide gel electrophoresis by immunoblotting, using 5% BSA blocking buffer and nitrocellulose membranes. The following antibodies were utilized: WNV NS3 (R&D BAF2907), Actin (C4; EMD MAB1501), STAT1 (CST 9172P), STING (CST D2PZF), pSTAT1 (Y701; CST 58D6), pSTING (CST D7C3S).

### Immunofluorescence

8E4 (or) 8x10^4 HFF cells were seeded onto glass coverslips in a 24-well plate. The following day, cells were infected with WNV at MOI = 1 or transfected with calf-thymus DNA (ctDNA; ISD) (Thermo Fisher, Waltham, MA, USA) at 3ug/ml final concentration using Lipofectamine 3000 and following the manufacturer’s protocol. 24h after WNV infection or 3h after ctDNA transfection cells were fixed with 4% paraformaldehyde for 15min at room temperature (RT). Cells were permeabilized with 0.1% Triton X-100 for 5min at RT. After blocking the cells for 30min with 3% BSA in PBS, immunofluorescent staining was performed overnight at 4°C with the following primary antibodies: rabbit-anti-STING (1:100, gifted by Glen Barber), mouse-anti-dsRNA (J2, 1:800, Scicons, Budapest, Hungary). Nuclei were counterstained with 4',6-Diamidino-2-Phenylindole, Dihydrochloride (DAPI, Thermo Fisher). Fluorophore coupled secondary antibodies (Thermo Fisher) were applied for 1h at RT. After washing with PBS samples were mounted onto glass slides using ProLong Gold (Thermo Fisher). Images were acquired with a Nikon Eclipse Ti confocal microscope equipped with a 60x oil immersion objective using the Nikon confocal software. Insets were captured with 4x enlargement of 600x images. Images were merged and processed using the Nikon confocal analysis software (Nikon, Melville, NY, USA).

### Flow cytometry

Mice were euthanized by isoflurane and perfused with 30-40mL PBS to ensure systemic removal of blood and residual intravascular leukocytes. **Spleens** were homogenized and single cell suspensions were treated with ACK lysis buffer to clear any remaining red blood cells, washed and resuspended in FACS buffer (1X PBS, 0.5% FBS). Cells were plated at 1E6 cells/well and stained for surface markers 15 minutes on ice. Cells were then fixed, permeabilized (Foxp3 Fixation/Permeabilization Concentrate and Diluent, Ebioscience) and stained intracellularly with antibodies for 30 minutes on ice. Flow cytometry was performed on a BD LSRII machine using BD FACSDiva software. Analysis was performed using FlowJo software. The following directly conjugated antibodies were used: B515-Foxp3, B710-CXCR3, G575-Ki67, G610-CTA-4, G666-CD127, G780-KLRG1, R660-NS4b Tet, R710-CD45, R780-CD44, UV395-CD8, UV730-CD3, V450-CD73, V610-CD4, V655-CD25, V510-live/dead. Cells were counted by hemocytometer using trypan blue exclusion. **Brains** were harvested into RPMI and mechanically suspended using a 70uM strainer. Each brain suspension was added to hypertonic Percoll to create a 30% Percoll solution, vortexed then centrifuged at 1250 rpm for 30 minutes at 4°C. Following centrifugation, the supernatant was aspirated and cell pellet treated with ACK lysis buffer to remove any residual red blood cells. Cells were then washed and filtered through a 70um nylon mesh to remove residual debris and resuspended in FACS buffer. Cells were counted using beads during FACS analysis. Cells were plated at 1E6 cells/well and stained for surface markers 15 minutes on ice. Cells were then fixed and extracellularly stained with antibodies for 30 minutes on ice. Flow cytometry was performed on a BD LSRII machine using BD FACSDiva software. Analysis was performed using FlowJo software. The following directly conjugated antibodies were used for Fig 7C–7I: FITC-CD19, PerCP-Cy5.5-CD103, PE-CD3e, PE-Cy7-CD4, APC-WNV Tetramer (NS4b), BV421-CD8a, BV510-CD45.2, BV786-CD44 (or) Fig 7K: V510-live/dead, R710-CD45, UV730-CD3, UV395-CD8, V610-CD4, V655-CD25, B515-Foxp3.

## Supporting information

S1 Fig (Supplemental to Figure 1): Terminal WT and STING-/- mice display increased GI pathology. A: Outcome of mock and WNV infected WT and STING-/- mice by study endpoint. Graph represents the outcome of each cohort as the percent of mock or WNV infected WT and STING-/- mice. Mice were retrospectively identified as either Terminal (T) or Survivors (S) for each cohort.
B: Gross pathology scores of the GI tract from necropsied mice. Mice were visually examined at necropsy and scored. Scores were assigned to each mouse ranging from 0 (normal GI tract) to 3 (grossly distended or aberrant morphology). n = 3–9 per condition; students t-test (unpaired). p = 0.05*.
C: Pathological analysis was performed on randomly selected representative mice. Sections of the GI were scored including sections from: 1) the duodenum and upper jejunum; 2) jejunum; 3) ileum; 4) cecum; 5) colon; 6) stomach. Graphed as the mean sum of all scores. n = 1–2 per condition.
D: Representative hematoxylin and eosin-stained small intestinal sections. Mock tissues were unremarkable with readily detectable myenteric ganglia (black ovals) and normal scant intestinal contents (inserts). Survivor mice have mild enteritis and minimal changes in the myenteric ganglia (ovals) with normal intestinal contents. In contrast, Terminal mice have myenteric ganglia with neuritis, degeneration and neuronal loss. There is bacterial overgrowth and exudative material within the intestinal contents (inserts at lower right of each image). In the STING-/- mice, there is readily observable vacuolation of the inner tunica muscularis (arrow), dilation of lacteals (asterisk) and intramucosal hemorrhage and lymphocytic and proliferative enteritis. All panels, original magnification 200X.
(TIF)

S2 Fig (Supplemental to Figure 5): Differential cytokine and chemokine profiles in WT and STING-/- mice. Serum luminex results with statistically significant differences in response to WNV infection between WT and STING-/- mice *in vivo*. Unpaired students t-test; p = 0.05*; p = 0.005*; p = 0.0005***).
(TIF)

S3 Fig (Supplemental to Figure 7): STING localization in the mouse brain. STING localization in the brain is centralized to the hind/mid-brain, hippocampus, primary motor-cortex and olfactory bulb in the brain. Square: midbrain/thalamus region. Oval: hindbrain (cerebellum and brain-stem). Image is from the Allen Institute for Brain Science. [Allen Mouse Brain Atlas]. Available from: [http://mouse.brain-map.org/gene/show/48353]. Images acquired using [Allen Brain Institute Brain Explorer 2]. Available from: [http://mouse.brain-map.org/static/brainexplorer].
(TIF)

## References

[1] Chow FC, Glaser CA. Emerging and reemerging neurologic infections. Neurohospitalist. 2014;4(4):173–84. 10.1177/1941874414540685 25360203 PMC4212420

[2] Johnson RT. Emerging viral infections of the nervous system. J Neurovirol. 2003;9(2):140–7. 10.1080/13550280390194091 . 12707845

[3] Petersen LR, Brault AC, Nasci RS. West Nile virus: review of the literature. JAMA. 2013;310(3):308–15. 10.1001/jama.2013.8042 23860989 PMC4563989

[4] Reimann CA, Hayes EB, DiGuiseppi C, Hoffman R, Lehman JA, Lindsey NP, et al Epidemiology of neuroinvasive arboviral disease in the United States, 1999–2007. Am J Trop Med Hyg. 2008;79(6):974–9. . 19052314

[5] Lindsey NP, Lehman JA, Staples JE, Fischer M, Division of Vector-Borne Diseases NCfE, Zoonotic Infectious Diseases CDC. West nile virus and other arboviral diseases—United States, 2013. MMWR Morb Mortal Wkly Rep. 2014;63(24):521–6. . 24941331 PMC5779373

[6] Petersen LR, Carson PJ, Biggerstaff BJ, Custer B, Borchardt SM, Busch MP. Estimated cumulative incidence of West Nile virus infection in US adults, 1999–2010. Epidemiol Infect. 2013;141(3):591–5. 10.1017/S0950268812001070 . 22640592 PMC9151873

[7] Sejvar JJ, Haddad MB, Tierney BC, Campbell GL, Marfin AA, Van Gerpen JA, et al Neurologic manifestations and outcome of West Nile virus infection. JAMA. 2003;290(4):511–5. 10.1001/jama.290.4.511 . 12876094

[8] Fine A, Layton M. Lessons from the West Nile viral encephalitis outbreak in New York City, 1999: implications for bioterrorism preparedness. Clin Infect Dis. 2001;32(2):277–82. 10.1086/318469 . 11170918

[9] Nash D, Mostashari F, Fine A, Miller J, O'Leary D, Murray K, et al The outbreak of West Nile virus infection in the New York City area in 1999. N Engl J Med. 2001;344(24):1807–14. 10.1056/NEJM200106143442401 . 11407341

[10] Griffin DE. Emergence and re-emergence of viral diseases of the central nervous system. Prog Neurobiol. 2010;91(2):95–101. Epub 2009/12/17. 10.1016/j.pneurobio.2009.12.003 20004230 PMC2860042

[11] Sejvar JJ, Curns AT, Welburg L, Jones JF, Lundgren LM, Capuron L, et al Neurocognitive and functional outcomes in persons recovering from West Nile virus illness. J Neuropsychol. 2008;2(Pt 2):477–99. . 19824176 10.1348/174866407x218312

[12] Carson PJ, Borchardt SM, Custer B, Prince HE, Dunn-Williams J, Winkelman V, et al Neuroinvasive disease and West Nile virus infection, North Dakota, USA, 1999–2008. Emerg Infect Dis. 2012;18(4):684–6. 10.3201/eid1804.111313 22469465 PMC3309699

[13] Zou S, Foster GA, Dodd RY, Petersen LR, Stramer SL. West Nile fever characteristics among viremic persons identified through blood donor screening. J Infect Dis. 2010;202(9):1354–61. 10.1086/656602 . 20874087

[14] Russo MV, McGavern DB. Immune Surveillance of the CNS following Infection and Injury. Trends Immunol. 2015;36(10):637–50. 10.1016/j.it.2015.08.002 26431941 PMC4592776

[15] Harris K. Gastrointestinal manifestations of acute West Nile virus infection in humans [Thesis ]. Texas Medical Center Dissertations (via ProQuest): The University of Texas; 2016.

[16] Armah HB, Wang G, Omalu BI, Tesh RB, Gyure KA, Chute DJ, et al Systemic distribution of West Nile virus infection: postmortem immunohistochemical study of six cases. Brain Pathol. 2007;17(4):354–62. Epub 2007/07/06. 10.1111/j.1750-3639.2007.00080.x . 17610522 PMC8095553

[17] Watson JT, Pertel PE, Jones RC, Siston AM, Paul WS, Austin CC, et al Clinical characteristics and functional outcomes of West Nile Fever. Ann Intern Med. 2004;141(5):360–5. Epub 2004/09/09. 10.7326/0003-4819-141-5-200409070-00010 . 15353427

[18] Leis AA, Stokic DS, Webb RM, Slavinski SA, Fratkin J. Clinical spectrum of muscle weakness in human West Nile virus infection. Muscle Nerve. 2003;28(3):302–8. Epub 2003/08/21. 10.1002/mus.10440 . 12929189

[19] Suthar MS, Diamond MS, Gale M Jr. West Nile virus infection and immunity. Nat Rev Microbiol. 2013;11(2):115–28. 10.1038/nrmicro2950 . 23321534

[20] Graham JB, Swarts JL, Lund JM. A Mouse Model of West Nile Virus Infection. Curr Protoc Mouse Biol. 2017;7(4):221–35. Epub 2017/12/21. 10.1002/cpmo.33 29261232 PMC5777180

[21] Graham JB, Swarts JL, Wilkins C, Thomas S, Green R, Sekine A, et al A Mouse Model of Chronic West Nile Virus Disease. PLoS Pathog. 2016;12(11):e1005996 Epub 2016/11/03. 10.1371/journal.ppat.1005996 27806117 PMC5091767

[22] Bingham J, Payne J, Harper J, Frazer L, Eastwood S, Wilson S, et al Evaluation of a mouse model for the West Nile virus group for the purpose of determining viral pathotypes. J Gen Virol. 2014;95(Pt 6):1221–32. Epub 2014/04/04. 10.1099/vir.0.063537-0 . 24694397

[23] Errett JS, Suthar MS, McMillan A, Diamond MS, Gale M Jr. The essential, nonredundant roles of RIG-I and MDA5 in detecting and controlling West Nile virus infection. J Virol. 2013;87(21):11416–25. 10.1128/JVI.01488-13 23966395 PMC3807316

[24] Suthar MS, Ma DY, Thomas S, Lund JM, Zhang N, Daffis S, et al IPS-1 is essential for the control of West Nile virus infection and immunity. PLoS Pathog. 2010;6(2):e1000757 10.1371/journal.ppat.1000757 20140199 PMC2816698

[25] Lazear HM, Daniels BP, Pinto AK, Huang AC, Vick SC, Doyle SE, et al Interferon-lambda restricts West Nile virus neuroinvasion by tightening the blood-brain barrier. Sci Transl Med. 2015;7(284):284ra59. Epub 2015/04/24. 10.1126/scitranslmed.aaa4304 25904743 PMC4435724

[26] Daffis S, Samuel MA, Keller BC, Gale M Jr., Diamond MS. Cell-specific IRF-3 responses protect against West Nile virus infection by interferon-dependent and -independent mechanisms. PLoS Pathog. 2007;3(7):e106 Epub 2007/08/07. 10.1371/journal.ppat.0030106 17676997 PMC1933455

[27] Cho H, Diamond MS. Immune responses to West Nile virus infection in the central nervous system. Viruses. 2012;4(12):3812–30. 10.3390/v4123812 23247502 PMC3528292

[28] Quicke KM, Suthar MS. The innate immune playbook for restricting West Nile virus infection. Viruses. 2013;5(11):2643–58. 10.3390/v5112643 24178712 PMC3856407

[29] Suthar MS, Brassil MM, Blahnik G, McMillan A, Ramos HJ, Proll SC, et al A systems biology approach reveals that tissue tropism to West Nile virus is regulated by antiviral genes and innate immune cellular processes. PLoS Pathog. 2013;9(2):e1003168 10.1371/journal.ppat.1003168 23544010 PMC3567171

[30] Fredericksen BL, Gale M Jr. West Nile virus evades activation of interferon regulatory factor 3 through RIG-I-dependent and -independent pathways without antagonizing host defense signaling. J Virol. 2006;80(6):2913–23. 10.1128/JVI.80.6.2913-2923.2006 16501100 PMC1395472

[31] Fredericksen BL, Keller BC, Fornek J, Katze MG, Gale M Jr. Establishment and maintenance of the innate antiviral response to West Nile Virus involves both RIG-I and MDA5 signaling through IPS-1. J Virol. 2008;82(2):609–16. 10.1128/JVI.01305-07 17977974 PMC2224571

[32] Keller BC, Fredericksen BL, Samuel MA, Mock RE, Mason PW, Diamond MS, et al Resistance to alpha/beta interferon is a determinant of West Nile virus replication fitness and virulence. J Virol. 2006;80(19):9424–34. 10.1128/JVI.00768-06 16973548 PMC1617238

[33] Samuel MA, Diamond MS. Alpha/beta interferon protects against lethal West Nile virus infection by restricting cellular tropism and enhancing neuronal survival. J Virol. 2005;79(21):13350–61. 10.1128/JVI.79.21.13350-13361.2005 16227257 PMC1262587

[34] Loo YM, Fornek J, Crochet N, Bajwa G, Perwitasari O, Martinez-Sobrido L, et al Distinct RIG-I and MDA5 signaling by RNA viruses in innate immunity. J Virol. 2008;82(1):335–45. 10.1128/JVI.01080-07 17942531 PMC2224404

[35] Lazear HM, Lancaster A, Wilkins C, Suthar MS, Huang A, Vick SC, et al IRF-3, IRF-5, and IRF-7 coordinately regulate the type I IFN response in myeloid dendritic cells downstream of MAVS signaling. PLoS Pathog. 2013;9(1):e1003118 10.1371/journal.ppat.1003118 23300459 PMC3536698

[36] Lazear HM, Pinto AK, Ramos HJ, Vick SC, Shrestha B, Suthar MS, et al Pattern recognition receptor MDA5 modulates CD8+ T cell-dependent clearance of West Nile virus from the central nervous system. J Virol. 2013;87(21):11401–15. 10.1128/JVI.01403-13 23966390 PMC3807324

[37] Suthar MS, Ramos HJ, Brassil MM, Netland J, Chappell CP, Blahnik G, et al The RIG-I-like receptor LGP2 controls CD8(+) T cell survival and fitness. Immunity. 2012;37(2):235–48. 10.1016/j.immuni.2012.07.004 22841161 PMC3910444

[38] Pinto AK, Ramos HJ, Wu X, Aggarwal S, Shrestha B, Gorman M, et al Deficient IFN signaling by myeloid cells leads to MAVS-dependent virus-induced sepsis. PLoS Pathog. 2014;10(4):e1004086 10.1371/journal.ppat.1004086 24743949 PMC3990718

[39] Pinto AK, Daffis S, Brien JD, Gainey MD, Yokoyama WM, Sheehan KC, et al A temporal role of type I interferon signaling in CD8+ T cell maturation during acute West Nile virus infection. PLoS Pathog. 2011;7(12):e1002407 Epub 2011/12/07. 10.1371/journal.ppat.1002407 22144897 PMC3228803

[40] Shrestha B, Diamond MS. Role of CD8+ T cells in control of West Nile virus infection. J Virol. 2004;78(15):8312–21. Epub 2004/07/16. 10.1128/JVI.78.15.8312-8321.2004 15254203 PMC446114

[41] Sitati EM, Diamond MS. CD4+ T-cell responses are required for clearance of West Nile virus from the central nervous system. J Virol. 2006;80(24):12060–9. Epub 2006/10/13. 10.1128/JVI.01650-06 17035323 PMC1676257

[42] Wang Y, Lobigs M, Lee E, Mullbacher A. CD8+ T cells mediate recovery and immunopathology in West Nile virus encephalitis. J Virol. 2003;77(24):13323–34. Epub 2003/12/04. 10.1128/JVI.77.24.13323-13334.2003 14645588 PMC296062

[43] Lanteri MC, O'Brien KM, Purtha WE, Cameron MJ, Lund JM, Owen RE, et al Tregs control the development of symptomatic West Nile virus infection in humans and mice. J Clin Invest. 2009;119(11):3266–77. 10.1172/JCI39387 19855131 PMC2769173

[44] Schoggins JW, MacDuff DA, Imanaka N, Gainey MD, Shrestha B, Eitson JL, et al Pan-viral specificity of IFN-induced genes reveals new roles for cGAS in innate immunity. Nature. 2014;505(7485):691–5. 10.1038/nature12862 24284630 PMC4077721

[45] Schoggins JW, MacDuff DA, Imanaka N, Gainey MD, Shrestha B, Eitson JL, et al Corrigendum: Pan-viral specificity of IFN-induced genes reveals new roles for cGAS in innate immunity. Nature. 2015;525(7567):144 10.1038/nature14555 . 26153856 PMC8323779

[46] You F, Wang P, Yang L, Yang G, Zhao YO, Qian F, et al ELF4 is critical for induction of type I interferon and the host antiviral response. Nat Immunol. 2013;14(12):1237–46. 10.1038/ni.2756 24185615 PMC3939855

[47] Ishikawa H, Barber GN. STING is an endoplasmic reticulum adaptor that facilitates innate immune signalling. Nature. 2008;455(7213):674–8. 10.1038/nature07317 18724357 PMC2804933

[48] Ishikawa H, Ma Z, Barber GN. STING regulates intracellular DNA-mediated, type I interferon-dependent innate immunity. Nature. 2009;461(7265):788–92. 10.1038/nature08476 19776740 PMC4664154

[49] Bhat N, Fitzgerald KA. Recognition of cytosolic DNA by cGAS and other STING-dependent sensors. Eur J Immunol. 2014;44(3):634–40. 10.1002/eji.201344127 24356864 PMC4621431

[50] Burdette DL, Vance RE. STING and the innate immune response to nucleic acids in the cytosol. Nat Immunol. 2013;14(1):19–26. 10.1038/ni.2491 . 23238760

[51] Ma Z, Damania B. The cGAS-STING Defense Pathway and Its Counteraction by Viruses. Cell Host Microbe. 2016;19(2):150–8. 10.1016/j.chom.2016.01.010 26867174 PMC4755325

[52] Maringer K, Fernandez-Sesma A. Message in a bottle: lessons learned from antagonism of STING signalling during RNA virus infection. Cytokine Growth Factor Rev. 2014;25(6):669–79. 10.1016/j.cytogfr.2014.08.004 25212897 PMC4330990

[53] Barber GN. STING: infection, inflammation and cancer. Nat Rev Immunol. 2015;15(12):760–70. Epub 2015/11/26. 10.1038/nri3921 26603901 PMC5004891

[54] Ni G, Ma Z, Damania B. cGAS and STING: At the intersection of DNA and RNA virus-sensing networks. PLoS Pathog. 2018;14(8):e1007148 Epub 2018/08/17. 10.1371/journal.ppat.1007148 30114241 PMC6095619

[55] Holm CK, Jensen SB, Jakobsen MR, Cheshenko N, Horan KA, Moeller HB, et al Virus-cell fusion as a trigger of innate immunity dependent on the adaptor STING. Nat Immunol. 2012;13(8):737–43. 10.1038/ni.2350 22706339 PMC3411909

[56] Holm CK, Rahbek SH, Gad HH, Bak RO, Jakobsen MR, Jiang Z, et al Influenza A virus targets a cGAS-independent STING pathway that controls enveloped RNA viruses. Nat Commun. 2016;7:10680 10.1038/ncomms10680 26893169 PMC4762884

[57] Aguirre S, Maestre AM, Pagni S, Patel JR, Savage T, Gutman D, et al DENV inhibits type I IFN production in infected cells by cleaving human STING. PLoS Pathog. 2012;8(10):e1002934 10.1371/journal.ppat.1002934 23055924 PMC3464218

[58] Nazmi A, Mukhopadhyay R, Dutta K, Basu A. STING mediates neuronal innate immune response following Japanese encephalitis virus infection. Sci Rep. 2012;2:347 10.1038/srep00347 22470840 PMC3317237

[59] Liu Y, Cherry S. Zika virus infection activates sting-dependent antiviral autophagy in the Drosophila brain. Autophagy. 2019;15(1):174–5. Epub 2018/09/28. 10.1080/15548627.2018.1528813 30260713 PMC6287696

[60] Chiu RW, Rainer TH, Lo YM. Circulating nucleic acid analysis: diagnostic applications for acute pathologies. Acta Neurochir Suppl. 2005;95:471–4. . 16463903 10.1007/3-211-32318-x_96

[61] de Rivero Vaccari JP, Dietrich WD, Keane RW. Activation and regulation of cellular inflammasomes: gaps in our knowledge for central nervous system injury. J Cereb Blood Flow Metab. 2014;34(3):369–75. 10.1038/jcbfm.2013.227 24398940 PMC3948131

[62] Liimatainen SP, Jylhava J, Raitanen J, Peltola JT, Hurme MA. The concentration of cell-free DNA in focal epilepsy. Epilepsy Res. 2013;105(3):292–8. 10.1016/j.eplepsyres.2013.03.005 . 23582956

[63] Tsai NW, Lin TK, Chen SD, Chang WN, Wang HC, Yang TM, et al The value of serial plasma nuclear and mitochondrial DNA levels in patients with acute ischemic stroke. Clin Chim Acta. 2011;412(5–6):476–9. 10.1016/j.cca.2010.11.036 . 21130757

[64] Field R, Campion S, Warren C, Murray C, Cunningham C. Systemic challenge with the TLR3 agonist poly I:C induces amplified IFNalpha/beta and IL-1beta responses in the diseased brain and exacerbates chronic neurodegeneration. Brain Behav Immun. 2010;24(6):996–1007. 10.1016/j.bbi.2010.04.004 20399848 PMC3334265

[65] Abdullah A, Zhang M, Frugier T, Bedoui S, Taylor JM, Crack PJ. STING-mediated type-I interferons contribute to the neuroinflammatory process and detrimental effects following traumatic brain injury. J Neuroinflammation. 2018;15(1):323 Epub 2018/11/23. 10.1186/s12974-018-1354-7 30463579 PMC6247615

[66] Ahn J, Barber GN. Self-DNA, STING-dependent signaling and the origins of autoinflammatory disease. Curr Opin Immunol. 2014;31:121–6. Epub 2014/12/03. 10.1016/j.coi.2014.10.009 . 25459004

[67] Gall A, Treuting P, Elkon KB, Loo YM, Gale M Jr., Barber GN, et al Autoimmunity initiates in nonhematopoietic cells and progresses via lymphocytes in an interferon-dependent autoimmune disease. Immunity. 2012;36(1):120–31. Epub 2012/01/31. 10.1016/j.immuni.2011.11.018 22284419 PMC3269499

[68] Sun L, Wu J, Du F, Chen X, Chen ZJ. Cyclic GMP-AMP synthase is a cytosolic DNA sensor that activates the type I interferon pathway. Science. 2013;339(6121):786–91. 10.1126/science.1232458 23258413 PMC3863629

[69] Wu J, Sun L, Chen X, Du F, Shi H, Chen C, et al Cyclic GMP-AMP is an endogenous second messenger in innate immune signaling by cytosolic DNA. Science. 2013;339(6121):826–30. 10.1126/science.1229963 23258412 PMC3855410

[70] Aarreberg LD, et al Interleukin-1β induces mitochondrial DNA release to activate innate immune signaling via cGAS-STING. Molecular Cell. 74(4):801–815. 2019 10.1016/j.molcel.2019.02.038 30952515 PMC6596306

[71] Liu S, Cai X, Wu J, Cong Q, Chen X, Li T, et al Phosphorylation of innate immune adaptor proteins MAVS, STING, and TRIF induces IRF3 activation. Science. 2015;347(6227):aaa2630. 10.1126/science.aaa2630 . 25636800

[72] Kelley TW, Prayson RA, Ruiz AI, Isada CM, Gordon SM. The neuropathology of West Nile virus meningoencephalitis. A report of two cases and review of the literature. Am J Clin Pathol. 2003;119(5):749–53. Epub 2003/05/23. 10.1309/PU4R-76JJ-MG1F-81RP . 12760295

[73] Tabula Muris 2019 [cited 2019 May 12]. Tmem173 (STING) cellular expression]. Available from: https://tabula-muris.ds.czbiohub.org/.

[74] Tabula Muris C, Overall c, Logistical c, Organ c, processing, Library p, et al Single-cell transcriptomics of 20 mouse organs creates a Tabula Muris. Nature. 2018;562(7727):367–72. 10.1038/s41586-018-0590-4 . 30283141 PMC6642641

[75] Allen Mouse Brain Atlas: Allen Brain Institute; 2004 [cited 2019 January 21]. Tmem173-/- (STING) in the mouse brain]. Available from: http://mouse.brain-map.org/gene/show/48353.

[76] Beckham JD, Pastula DM, Massey A, Tyler KL. Zika Virus as an Emerging Global Pathogen: Neurological Complications of Zika Virus. JAMA Neurol. 2016 10.1001/jamaneurol.2016.0800 . 27183312 PMC5087605

[77] Carod-Artal FJ. Epidemiology and neurological complications of infection by the Zika virus: a new emerging neurotropic virus. Rev Neurol. 2016;62(7):317–28. . 26988170

[78] Suen WW, Prow NA, Setoh YX, Hall RA, Bielefeldt-Ohmann H. End-point disease investigation for virus strains of intermediate virulence as illustrated by flavivirus infections. J Gen Virol. 2016;97(2):366–77. 10.1099/jgv.0.000356 . 26614392

[79] Thackray LB, Handley SA, Gorman MJ, Poddar S, Bagadia P, Briseno CG, et al Oral Antibiotic Treatment of Mice Exacerbates the Disease Severity of Multiple Flavivirus Infections. Cell Rep. 2018;22(13):3440–53 e6. Epub 2018/03/29. 10.1016/j.celrep.2018.03.001 29590614 PMC5908250

[80] White JP, Xiong S, Malvin NP, Khoury-Hanold W, Heuckeroth RO, Stappenbeck TS, et al Intestinal Dysmotility Syndromes following Systemic Infection by Flaviviruses. Cell. 2018;175(5):1198–212 e12. 10.1016/j.cell.2018.08.069 30293866 PMC6309989

[81] Luksch H, Stinson WA, Platt DJ, Qian W, Kalugotla G, Miner CA, et al STING-associated lung disease in mice relies on T cells but not type I interferon. J Allergy Clin Immunol. 2019 10.1016/j.jaci.2019.01.044 . 30772497 PMC6612314

[82] Ramos HJ, Gale M Jr. RIG-I like receptors and their signaling crosstalk in the regulation of antiviral immunity. Curr Opin Virol. 2011;1(3):167–76. 10.1016/j.coviro.2011.04.004 21949557 PMC3177754

[83] Ramos HJ, Lanteri MC, Blahnik G, Negash A, Suthar MS, Brassil MM, et al IL-1beta signaling promotes CNS-intrinsic immune control of West Nile virus infection. PLoS Pathog. 2012;8(11):e1003039 10.1371/journal.ppat.1003039 23209411 PMC3510243

[84] Sauer JD, Sotelo-Troha K, von Moltke J, Monroe KM, Rae CS, Brubaker SW, et al The N-ethyl-N-nitrosourea-induced Goldenticket mouse mutant reveals an essential function of Sting in the in vivo interferon response to Listeria monocytogenes and cyclic dinucleotides. Infect Immun. 2011;79(2):688–94. Epub 2010/11/26. 10.1128/IAI.00999-10 21098106 PMC3028833

[85] Sharples SA, Koblinger K, Humphreys JM, Whelan PJ. Dopamine: a parallel pathway for the modulation of spinal locomotor networks. Front Neural Circuits. 2014;8:55 Epub 2014/07/02. 10.3389/fncir.2014.00055 24982614 PMC4059167

[86] Muller DM, Pender MP, Greer JM. A neuropathological analysis of experimental autoimmune encephalomyelitis with predominant brain stem and cerebellar involvement and differences between active and passive induction. Acta Neuropathol. 2000;100(2):174–82. . 10963365 10.1007/s004019900163

[87] Pierson ER, Stromnes IM, Goverman JM. B cells promote induction of experimental autoimmune encephalomyelitis by facilitating reactivation of T cells in the central nervous system. J Immunol. 2014;192(3):929–39. 10.4049/jimmunol.1302171 24367024 PMC3934009

[88] Palle P, Ferreira FM, Methner A, Buch T. The more the merrier? Scoring, statistics and animal welfare in experimental autoimmune encephalomyelitis. Lab Anim. 2016;50(6):427–32. Epub 2016/12/03. 10.1177/0023677216675008 . 27909192

[89] Miller SD, Karpus WJ. Experimental autoimmune encephalomyelitis in the mouse. Curr Protoc Immunol. 2007;Chapter 15:Unit 15 1. 10.1002/0471142735.im1501s77 18432984 PMC2915550

[90] Treuting PM, Snyder JM. Mouse Necropsy. Curr Protoc Mouse Biol. 2015;5(3):223–33. Epub 2015/09/04. 10.1002/9780470942390.mo140296 . 26331757

[91] Daniels BP, Snyder AG, Olsen TM, Orozco S, Oguin TH 3rd, Tait SWG, et al RIPK3 Restricts Viral Pathogenesis via Cell Death-Independent Neuroinflammation. Cell. 2017;169(2):301–13 e11. Epub 2017/04/04. 10.1016/j.cell.2017.03.011 28366204 PMC5405738

[92] Klein RS, Lin E, Zhang B, Luster AD, Tollett J, Samuel MA, et al Neuronal CXCL10 directs CD8+ T-cell recruitment and control of West Nile virus encephalitis. J Virol. 2005;79(17):11457–66. Epub 2005/08/17. 10.1128/JVI.79.17.11457-11466.2005 16103196 PMC1193600

[93] Brien JD, Lazear HM, Diamond MS. Propagation, quantification, detection, and storage of West Nile virus. Curr Protoc Microbiol. 2013;31:15D 3 1-D 3 8. Epub 2014/02/11. 10.1002/9780471729259.mc15d03s31 . 24510289

