# Supplementary figures and images for: STING is required for host defense against neuropathological West Nile virus infection
Source: PLoS Pathog. 2019 Aug 15;15(8):e1007899. doi: 10.1371/journal.ppat.1007899 (PMC6695101; doi:10.1371/journal.ppat.1007899)

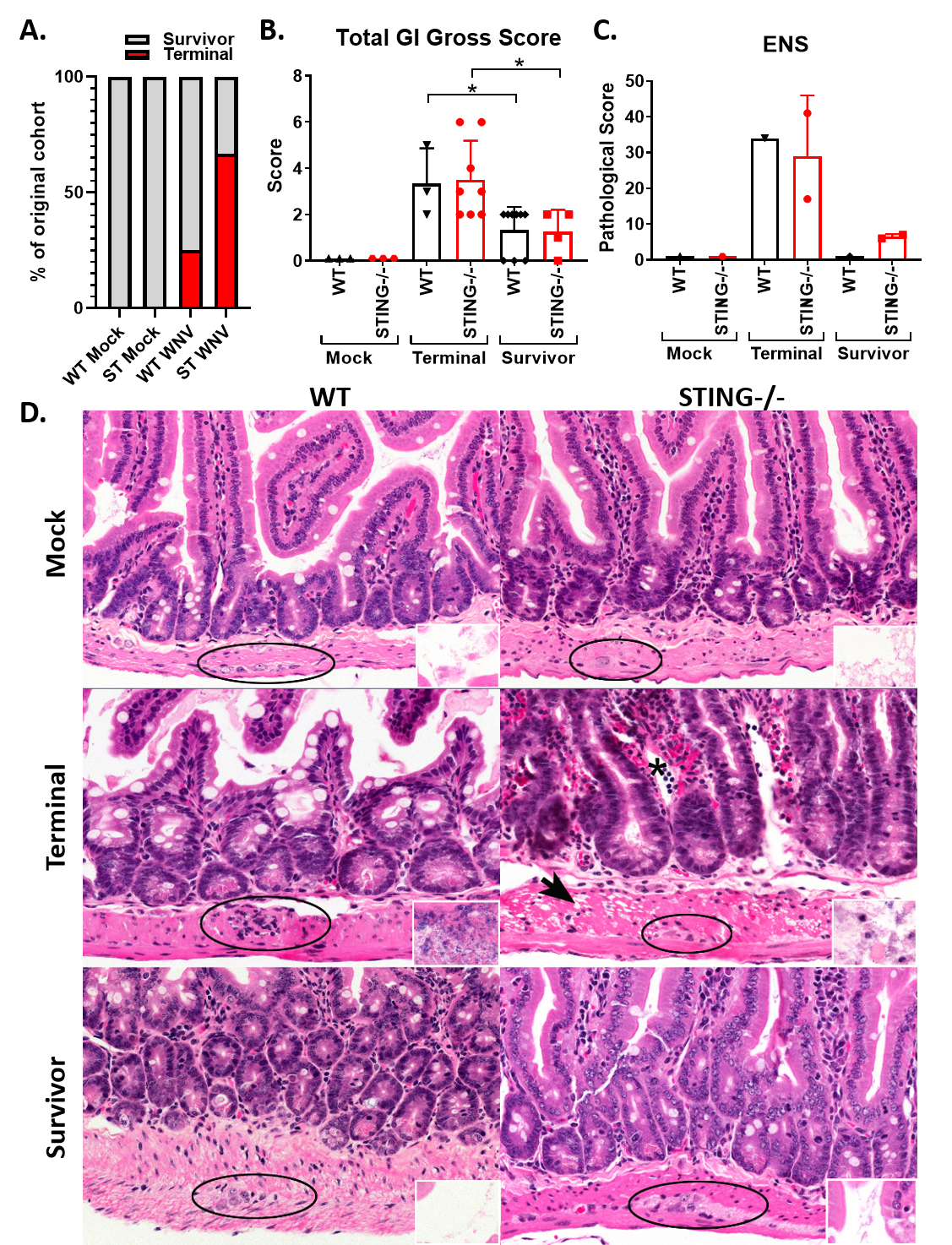

Supplement: S1 Fig — A: Outcome of mock and WNV infected WT and STING-/- mice by study endpoint. Graph represents the outcome of each cohort as the percent of mock or WNV infected WT and STING-/- mice. Mice were retrospectively identified as either Terminal (T) or Survivors (S) for each cohort. B: Gross pathology scores of the GI tract from necropsied mice. Mice were visually examined at necropsy and scored. Scores were assigned to each mouse ranging from 0 (normal GI tract) to 3 (grossly distended or aberrant morphology). n = 3–9 per condition; students t-test (unpaired). p = 0.05*. C: Pathological analysis was performed on randomly selected representative mice. Sections of the GI were scored including sections from: 1) the duodenum and upper jejunum; 2) jejunum; 3) ileum; 4) cecum; 5) colon; 6) stomach. Graphed as the mean sum of all scores. n = 1–2 per condition. D: Representative hematoxylin and eosin-stained small intestinal sections. Mock tissues were unremarkable with readily detectable myenteric ganglia (black ovals) and normal scant intestinal contents (inserts). Survivor mice have mild enteritis and minimal changes in the myenteric ganglia (ovals) with normal intestinal contents. In contrast, Terminal mice have myenteric ganglia with neuritis, degeneration and neuronal loss. There is bacterial overgrowth and exudative material within the intestinal contents (inserts at lower right of each image). In the STING-/- mice, there is readily observable vacuolation of the inner tunica muscularis (arrow), dilation of lacteals (asterisk) and intramucosal hemorrhage and lymphocytic and proliferative enteritis. All panels, original magnification 200X. (TIF) [file ppat.1007899.s001.tif]

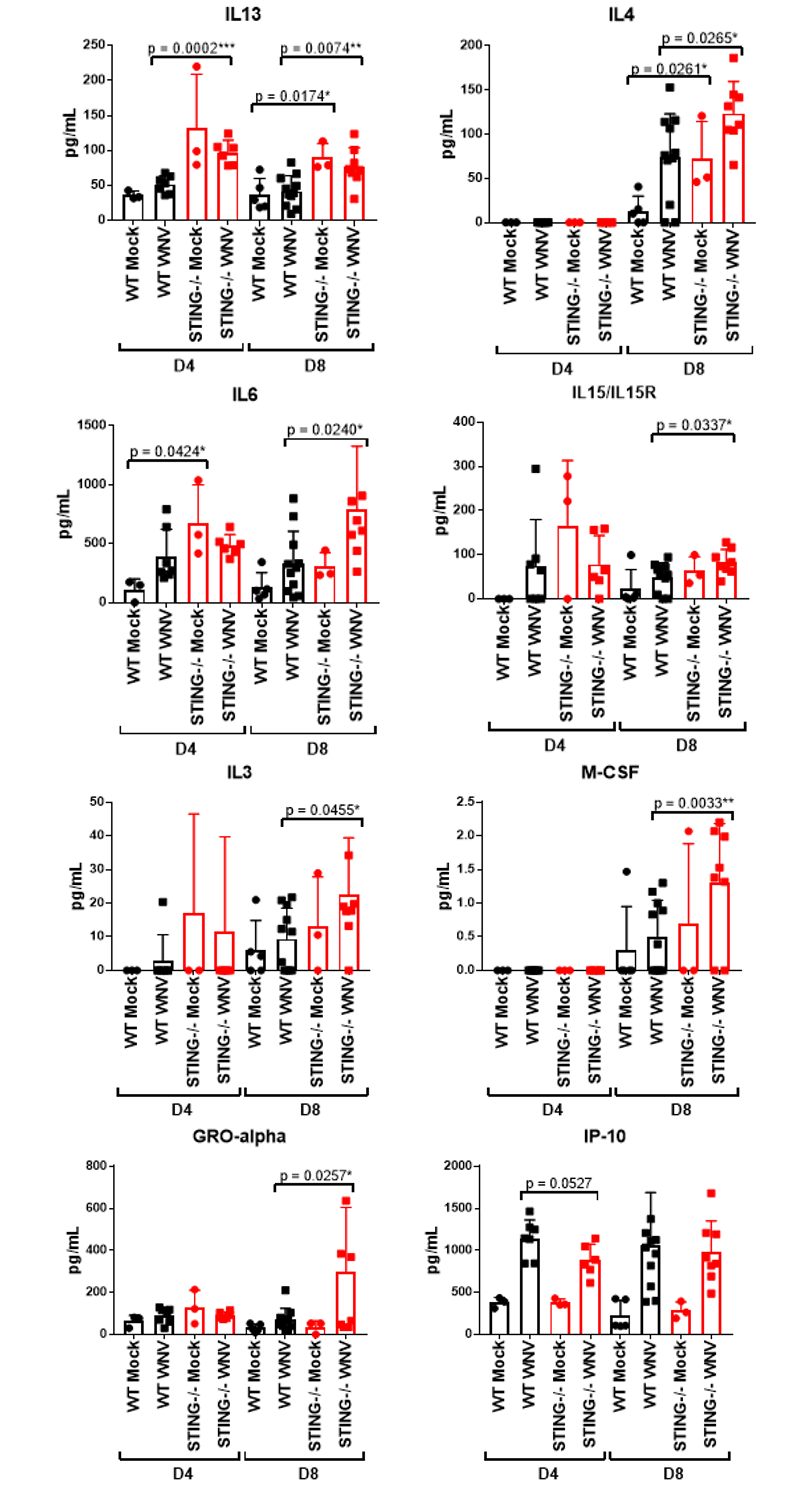

Supplement: S2 Fig — Serum luminex results with statistically significant differences in response to WNV infection between WT and STING-/- mice in vivo. Unpaired students t-test; p = 0.05*; p = 0.005*; p = 0.0005***). (TIF) [file ppat.1007899.s002.tif]

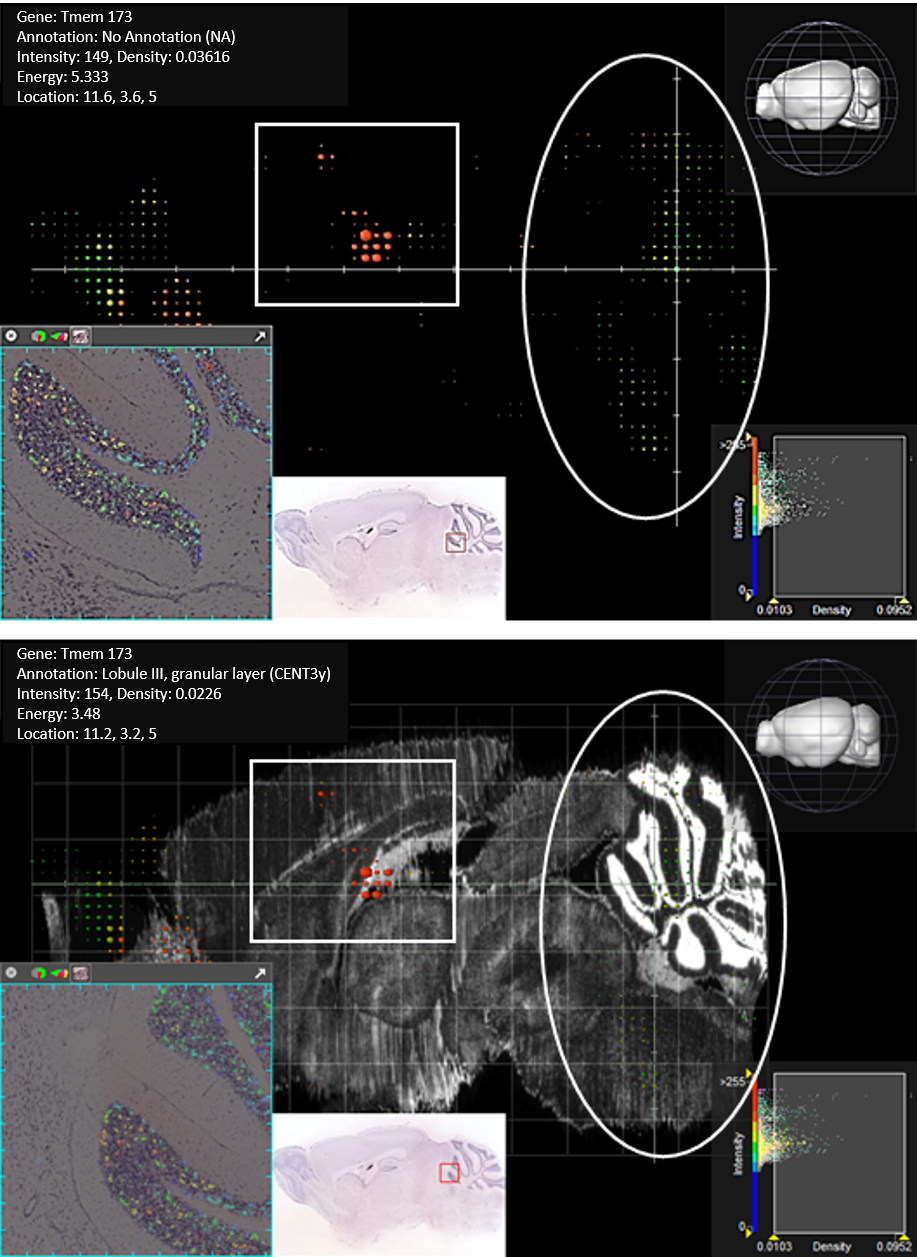

Supplement: S3 Fig — STING localization in the brain is centralized to the hind/mid-brain, hippocampus, primary motor-cortex and olfactory bulb in the brain. Square: midbrain/thalamus region. Oval: hindbrain (cerebellum and brain-stem). Image is from the Allen Institute for Brain Science. [Allen Mouse Brain Atlas]. Available from: [http://mouse.brain-map.org/gene/show/48353]. Images acquired using [Allen Brain Institute Brain Explorer 2]. Available from: [http://mouse.brain-map.org/static/brainexplorer]. (TIF) [file ppat.1007899.s003.tif]
